# A reverse metabolic approach to weaning: in silico identification of immune-beneficial infant gut bacteria, mining their metabolism for prebiotic feeds and sourcing these feeds in the natural product space

**DOI:** 10.1186/s40168-018-0545-x

**Published:** 2018-09-21

**Authors:** Samanta Michelini, Biju Balakrishnan, Silvia Parolo, Alice Matone, Jane A. Mullaney, Wayne Young, Olivier Gasser, Clare Wall, Corrado Priami, Rosario Lombardo, Martin Kussmann

**Affiliations:** 1 The Microsoft Research–University of Trento Centre for Computational and Systems Biology, Rovereto, Italy; 2 The Liggins Institute, the University of Auckland, Auckland, New Zealand; 3 AgResearch, Food & Bio-based Products, Palmerston North, New Zealand; 4 Riddet Institute, Palmerston North, New Zealand; 5 Malaghan Institute of Medical Research, Wellington, New Zealand; 6 Discipline of Nutrition, School of Medical Science, University of Auckland, Auckland, New Zealand; 7 Department of Computer Science, University of Pisa, Pisa, Italy; 8 National Science Challenge “High Value Nutrition”, Auckland, New Zealand

**Keywords:** Infant gut microbiome, Prebiotic, Probiotic, Reverse ecology, Infection, Knowledge extraction, Complementary feeding, Systems biology

## Abstract

**Background:**

Weaning is a period of marked physiological change. The introduction of solid foods and the changes in milk consumption are accompanied by significant gastrointestinal, immune, developmental, and microbial adaptations. Defining a reduced number of infections as the desired health benefit for infants around weaning, we identified in silico (i.e., by advanced public domain mining) infant gut microbes as potential deliverers of this benefit. We then investigated the requirements of these bacteria for exogenous metabolites as potential prebiotic feeds that were subsequently searched for in the natural product space.

**Results:**

Using public domain literature mining and an in silico reverse metabolic approach, we constructed probiotic-prebiotic-food associations, which can guide targeted feeding of immune health-beneficial microbes by weaning food; analyzed competition and synergy for (prebiotic) nutrients between selected microbes; and translated this information into designing an experimental complementary feed for infants enrolled in a pilot clinical trial (http://www.nourishtoflourish.auckland.ac.nz/).

**Conclusions:**

In this study, we applied a benefit-oriented microbiome research strategy for enhanced early-life immune health. We extended from “classical” to molecular nutrition aiming to identify nutrients, bacteria, and mechanisms that point towards targeted feeding to improve immune health in infants around weaning. Here, we present the systems biology-based approach we used to inform us on the most promising prebiotic combinations known to support growth of beneficial gut bacteria (“probiotics”) in the infant gut, thereby favorably promoting development of the immune system.

**Electronic supplementary material:**

The online version of this article (10.1186/s40168-018-0545-x) contains supplementary material, which is available to authorized users.

## Background

The human body is host to 10^14^ resident microorganisms (bacteria, viruses, fungi, and protozoa) [1] that live in synergy with human cells influencing health outcomes across lifespan [2]. Depending on micro-environmental conditions, each body site is colonized by specific microbial communities shaped by co-evolution with the host. The majority of those microbes live in our gastrointestinal (GI) tract [3]. At birth, the pristine infant gut is rapidly colonized by millions of bacteria, some beneficial, some not. The first microbial inoculum is acquired at birth with differences in both diversity and abundance driven by host genetics, prenatal and maternal factors, such as the delivery mode, i.e., cesarean or vaginal [4]. The newborn’s microbiome is not yet stable and varies inter-individually [5]. The changes occur as a consequence of exposure to different environmental and health/disease conditions, dietary patterns, and also exposure to antibiotics. A diverse and balanced gut microbiome provides benefits to the host through affecting several physiological processes [6] ranging from maturation of the immune system, regulation of host metabolism, response to nutrition, metabolism of bioactive molecules and drugs [6], biosynthesis of vitamins and amino acids, and absorption of iron [2].

In the first 1000 days of life, the development of the infant’s microbiome is intimately tied to maturation of the immune system [7]. It has long been hypothesized that microbial exposure in early life has a protective effect on the newborn’s health and can influence the health outcomes later in life [4]. The gut microbiota is required for maturation and maintenance of the immune system and has a role in modulation of the immune system. For example, the immune system-microbiota interaction enables induction of protective responses to pathogens and the maintenance of regulatory pathways involved in the tolerance to innocuous antigens [8].

While several studies support the role of diet in shaping the gut microbial community in adults, only a few focused on understanding the contribution of complementary foods during the weaning period to the modulation of the infant gut microbiome [9]. Weaning is a phase of marked physiological change. The introduction of solid foods and the changes in milk consumption trigger significant GI tract, immune and developmental adaptations. Weaning also exposes infants to non-digestible carbohydrates and provides new substrates for the microbial gut community with resulting growth and dominance of some taxa, such as *Bacteroides* [10], and a reduction of others, such as bifidobacteria, enterobacteria, and some *Clostridium* spp. [9]. Hence, infant nutrition, especially in the complementary feeding period, exerts profound health impacts and careful dietary interventions during weaning can support the development of both a healthy microbiome and immune system, thereby improving child growth and development [11].

We report on an innovative and integrated pipeline developed to support complementary feeding design with the identification of essential metabolites required by beneficial and protective bacteria (“prebiotic feeds for probiotic bacteria”). After a comprehensive capture of the literature on the infant gut microbiome, the pipeline drives the selection of candidate immune-protective infant GI bacteria. By applying an in silico reverse metabolic approach, which uses the microbial metabolic network to infer its nutritional requirements without prior information, we mined the microbial metabolism to identify exogenous compounds required by candidate bacteria, which were sourced from the chemical composition of foods in order to enable identification of prebiotic ingredients naturally present in food and which can be given safely to infants during weaning. Our systems biology approach has informed the design and development of a complementary prebiotic feeding to nourish the microbiota that supports the immune system of infants and that promotes protection against common infections (http://www.nourishtoflourish.auckland.ac.nz/). Essentially, our methodology addresses and answers the following questions: (i) which immune protection-beneficial infant GI bacteria are present around and after weaning?; (ii) can we interrogate bacterial metabolism by identifying key enzymes involved in exogenous metabolite conversion and what do these preferred bacteria predominantly feed (“prebiotics”)?; (iii) is there an interaction for these metabolites between the selected bacteria and are essential nutrients cross-fed between bacteria?; and (iv) where and how can “prebiotic” whole foods or food components be sourced from the food chain?

## Results

### Identification of immune-protective bacteria (question i)

**Fig. 1:**
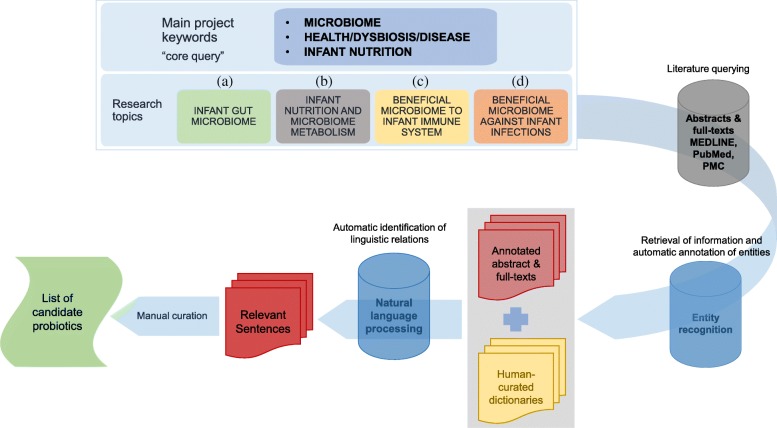
Public domain mining workflow for identifying intestinal bacteria, known or suggested to exert beneficial effects on the infant immune system. The main scientific concepts for the project were categorized into four research topics: (**a**) infant gut microbiome, (**b**) infant nutrition and microbiome metabolism, (**c**) beneficial bacteria supporting immune system development in infants, and (**d**) beneficial bacteria preventing infections in infants. For each topic, a set of keywords was identified to retrieve all relevant publications from MEDLINE, PubMed, and PubMed Central. All relevant terms, belonging to the entities: diseases, genes, chemicals, and species, were annotated. Specific *dictionaries* were then created selecting all terms relevant for the present study. Sentences showing a relationship between *dictionary* concepts were extracted from the texts through natural language processing. The output is a list of sentences with the following related pieces of information: PubMed ID, journal section, terms in the relationships and, for each dictionary, the list of terms found

The core query (Fig. 1) yielded a total of 3673 unique PubMed IDs by searching titles and abstracts and specifically 1657 PubMed IDs for infant gut microbiome, 608 PubMed IDs for infant nutrition and microbiome metabolism, 583 PubMed IDs for beneficial bacteria supporting immune system development in infant, and 825 PubMed IDs for beneficial bacteria preventing infections in infants.

**Fig. 2:**
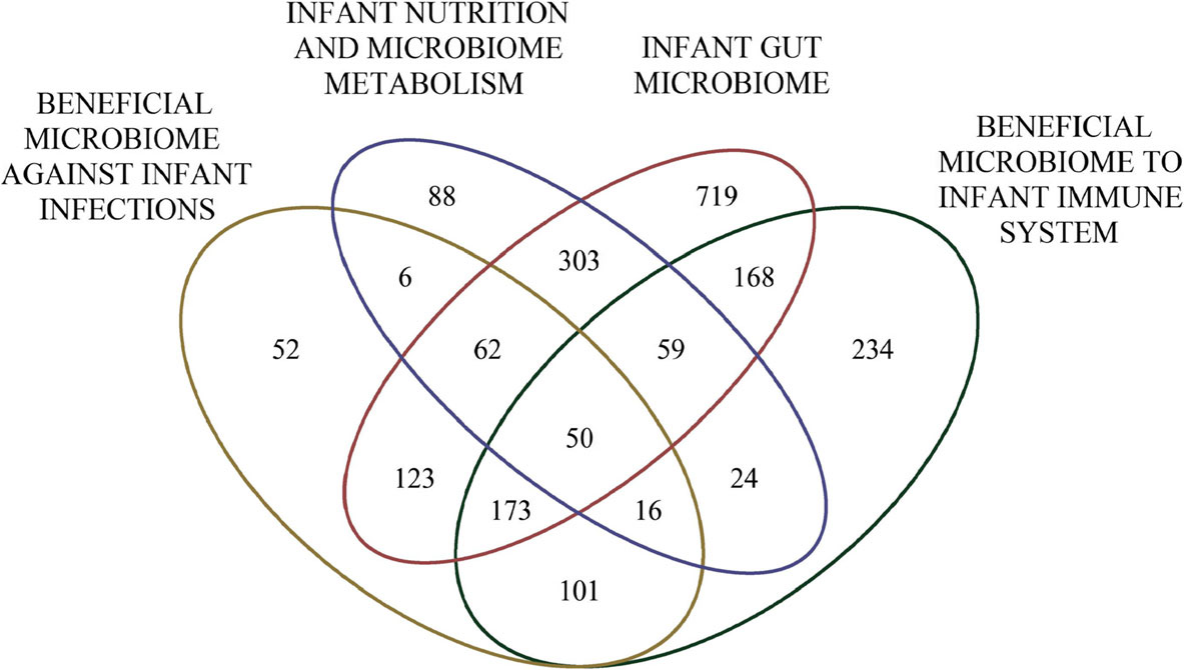
Venn diagram of PubMed IDs showing the number of abstracts found for each research topic and the overlap between them

The PubMed IDs shared by single or multiple research topics are shown in the Venn diagram of Fig. 2.

Fifty PubMed IDs were common to all search criteria. Available full texts (or abstracts when full texts were not available) were automatically annotated for cited biological entities to build dictionaries of relevant terms for the sentence extraction. As detailed in the Methods section, for each of the queries (a), (b), (c) and (d), the pipeline extracted relationships between each pair of the four dictionaries and co-mentions of more than two different dictionaries (Table 1), that were filtered to obtain the final list of sentences (Additional files 1 and 2).

**Table 1:** Combinations of the dictionaries used in text mining to extract sentences showing relationships/co-mentions from public domain literature

Relationship	No. of PubMed IDs	No. of Sent.	Co-mention	No. of PubMed IDs	No. of Sent.
gut-microbes | immune-related	89 (208)	172 (427)	gut-microbes | immune-related | infection-disease	42 (48)	66 (74)
gut-microbes | infection-disease	108 (316)	185 (564)	gut-microbes | immune-related | chemical-related	19 (74)	25 (132)
gut-microbes | chemical-related	0 (312)	0 (842)	gut-microbes | infection-disease | chemical-related	25 (58)	31 (75)
immune-related | infection-disease	0 (284)	0 (283)	immune-related | infection-disease | chemical-related	3 (7)	3 (49)
immune-related | chemical-related	0 (185)	0 (185)	gut-microbes | immune-related | infection-disease | chemical-related	2 (7)	2 (9)
infection-disease | chemical-related	0 (155)	0 (156)			

We constructed four dictionaries based on four concepts as described in the “Methods” section. The “Relationship” column shows the combinations of two dictionaries from the two concepts that were analyzed by natural language processing for extracting sentences containing a *linguistic relation* among both (Additional file 1). The “Co-mention” column shows the combinations of more than two concepts that were analyzed by natural language processing for extracting sentences *mentioning* those concepts (Additional file 2). For both “Relationship” and “Co-mention,” the table gives the number of PubMed IDs and the number of sentences that are extracted with the text mining pipeline after filtering for publication year (> 1999), human and mice studies, presence of microbial species, exclusion of pathogens and microbial genus names as described in the “Methods” section. Values before filtering are presented in parenthesis

The list of all microbes cited in the extracted sentences for both relationships and co-mentions was then manually curated (Additional files 1 and 2, “*-species” sheets). Examples of relevant sentences and associated PubMed IDs from which the relevant bacterial species are extracted are given in Additional file 3: Table S1.

We firstly concentrated our efforts on outcomes from queries (c) beneficial bacteria supporting immune system development in infants and (d) beneficial bacteria preventing infections in infants. Our pipeline recognized 27 unique species names from the relations of both queries (Additional file 1, “*-species” sheets). Among those lists, the most frequent species were *Lactobacillus rhamnosus* (appearing in 67 and 72 of the 223 and 235 sentences extracted for queries (c) and (d) respectively) and *Lactobacillus casei* in query (c) (37/223) and *Lactobacillus acidophilus* (33/235) in query (d). Queries (a) infant gut microbiome and (b) infant nutrition and microbiome metabolism, which were expected to collect ecological information on the infant microbiome and metabolism, found again *L*. *rhamnosus* as the most cited species, 77/260 and 15/60 sentences from query (a) and (b) respectively, and *L*. *acidophilus* (35/260) and *Bifidobacterium breve* (12/60) from query (b) as they are known to be infant gut commensals. As expected, co-mention analysis produced weaker microbial results after manual curation (Additional file 2, “*-species” sheets); we obtained a total of 22 species names from query (c) and 24 from query (d) that confirm the main outcomes from relationships. Based on those lists, the putative probiotic strains implicated in immune defense against infections in infants were evaluated and selected: 14 of the most promising bacteria belonged to bifidobacteria and lactobacilli. Based on our text mining results and from reports of their increasing abundance in healthy babies from 4 to 12 months of age [12], *Akkermansia muciniphila* and *Faecalibacterium prausnitzii* were also selected as potential “probiotics” in our study*.* General literature mining revealed the presence of *Bifidobacterium angulatum* in Japanese children at 3 years of age [13], and Haarman and Knol [14] found this species harbored by German infants between 28 and 90 days of age, and possible relations with feeding mode: *B*. *angulatum* seems to be associated with breast-feeding rather than formula feeding. *Bifidobacterium catenulatum* was also reported in mother-infant pairs [15, 16]. Both these strains were able to modulate cytokine secretion when tested in vitro in peripheral blood mononuclear cells [17]. We therefore included *B. angulatum* and *B. catenulatum* into our selected microbial list to further analyze their interactions with other bacteria.

### Identification of exogenous prebiotics for the selected microorganisms (question ii)

For each strain of the 18 selected strains (Table 2), which for simplicity will be referred to as a “community” the metabolic model was retrieved from the Virtual Metabolic Human (VMH) database.

**Table 2:** Putative protective probiotic strains selected. The selection aims to mimic an immune-enhancing and infection-protective probiotic community in the infant gut. These strains were subjected to subsequent network metabolic network analysis. The “Exogenous metabolite #” column shows the number of exogenous compounds required by each bacterium

Putative probiotic	Exogenous metabolite #
*Bifidobacterium adolescentis* ATCC 15703	251
*Bifidobacterium angulatum* DSM 20098	268
*Bifidobacterium animalis* subsp. *lactis* BB 12	248
*Bifidobacterium bifidum* BGN4	255
*Bifidobacterium breve* UCC2003 NCIMB8807	258
*Bifidobacterium catenulatum* DSM 16992	274
*Bifidobacterium longum* subsp. *infantis* ATCC 15697	295
*Bifidobacterium longum* subsp. *longum* CCUG 52486	289
*Bifidobacterium pseudocatenulatum* DSM 20438	283
*Lactobacillus acidophilus* NCFM	220
*Lactobacillus casei* subsp. *casei* BL23	313
*Lactobacillus fermentum* IFO 3956	265
*Lactobacillus paracasei* subsp*. paracasei* ATCC 25302	305
*Lactobacillus plantarum* WCFS1	308
*Lactobacillus reuteri* SD2112 ATCC 55730	261
*Lactobacillus rhamnosus* GG ATCC 53103	276
*Akkermansia muciniphila* ATCC BAA 835	208
*Faecalibacterium prausnitzii* M21 2	275

The topology of reconstructed metabolic networks was analyzed with NetSeed to identify exogenously acquired metabolites, and the nutrient profile of each strain was inferred (Additional file 4). We noted oxygen (O_2_) was included in the requirements of anaerobic bacteria and, while there are studies reporting exogenous oxygen uptake by *Bifidobacterium* spp. [18, 19], this might be linked to the VMH model reconstruction (see the “Methods” section). Table 2 shows the number of metabolites identified as exogenous on a network topology basis and thus putatively corresponding to essential nutrients required by each strain.

Our in silico study of this microbial community of probiotics identified a total of 632 unique exogenous metabolites which are potentially required for growth under normal circumstances [20]. Among them, 62 are essential for all strains, 200 are required by single microbes in the community, and 398 are shared between different strains. *L*. *casei* subsp. *casei* BL23 requires the most of exogenous metabolites (313), while *A*. *muciniphila* ATCC BAA 835 requires only 208 such essential metabolites. Interestingly, *F*. *prausnitzii* M21 2 shows highly distinct nutritional requirements compared to the other microorganisms: it needs 99 compounds for its metabolism that are not shared with other members in the community. On the other hand, lactobacilli share the majority of their necessary metabolites and only 5 are specific for the lactobacilli species considered in this study.

Grouping probiotics by genera, *Bifidobacterium* spp. require 398 nutrients for their growth; among these, 177 are necessary for all nine strains selected in this study, while 52 are strain-specific. Four hundred eighty-six exogenous compounds were detected for *Lactobacillus* species; 107 are present in the set seed of all seven strains, while 92 appear to be strain-specific. Five hundred seventy-six unique metabolites are required by the bifidobacteria-lactobacilli community; 75 are essential for all strains, and 73 are strain-specific.

### Cooperative metabolic bacteria-bacteria and bacteria-host interactions (question iii)

**Fig. 3:**
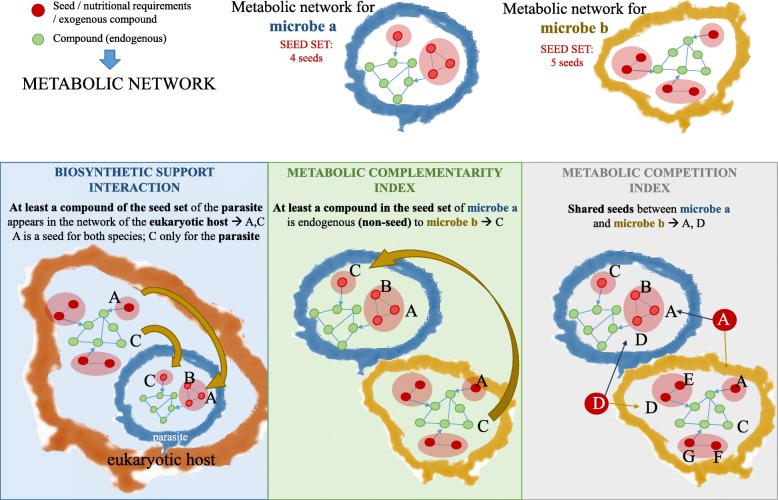
For two interacting species, the metabolic network is shown; nodes are metabolites and edges connect substrates to products. Substrates are colored in red, whereas potential products are in green. The *support* gives an example of the capacity of a potential eukaryotic host (orange) to meet the metabolic requirements of a microbe (blue). *Complementarity* represents exogenously acquired compounds in one species (blue) that are found in the metabolic network, although not as its feed, of the other species (yellow). *Competition* shows metabolic competition between two microbes (blue and yellow) for the same essential metabolites

Based on graph theory-based methods [21], both host-microbe and microbe-microbe cooperation was analyzed for each pair of selected probiotics. The cooperative and competitive interactions within the selected microbial community were investigated adopting a reverse ecology approach. We computed the *biosynthetic support scores* for each microbe-human host pair and the *metabolic complementarity indices* as well as the *metabolic competition indices* for each microbe-microbe pair in our community. Figure 3 graphically depicts the characteristics of these three scores that are abbreviated as *support*, *complementarity*, and *competition*, respectively, in the remaining of the text, for reasons of simplicity.

All probiotic species identified in this study exhibit low *support* scores versus the human host, ranging from 0.330 to 0.522, confirming that they are indeed gut commensals rather than parasites. Highest support scores were obtained for *A*. *muciniphila* and *L*. *acidophilus* (0.502 and 0.522), as they are evolved to live in the gut and hence have higher dependence on the host for provision of exogenous metabolites.

Our microbial community (lactobacilli, bifidobacteria, *A*. *muciniphila*, *F*. *prausnitzii*, *B*. *angulatum*, and *B*. *catenulatum*) is characterized by low *complementarity* indices. *Lactobacillus paracasei* subsp*. paracasei* is the most “supported” bacterium, while the “altruistic” *F. prausnitzii* complements most of the community members and is the least “supported.” *Bifidobacterium* is the taxon showing the lowest complementarity (0.064 for one pair, mean value 0.190 for bifidobacteria group), while lactobacilli show the highest values (0.243 for one specific pair, mean value 0.127 for lactobacilli group). We infer that *B*. *breve* and *Bifidobacterium bifidum* is the least complementing pair within bifidobacteria (complementarity 0.006/0.069), whereas *L*. *rhamnosus* and *L*. *casei* subsp. *casei* and *L*. *paracasei* subsp*. paracasei* show a low potential for syntrophy within lactobacilli (complementarity values 0.010/0.150, 0.010/162, respectively). The lactobacilli species most likely to complement each other are *L. acidophilus* and *Lactobacillus reuteri* as they show the highest complementarity scores (0.243 and 0.164, respectively).

Overall, we can assume complementarity between lactobacilli and bifidobacteria. Several of our in silico predictions on microbe metabolic cooperativity are supported by experimental studies: (i) for example, our finding of *B*. *breve* of enhancing *L*. *paracasei* subsp. *paracasei* (complementarity score 0.239) confirms results from in vitro GI models [22]. (ii) *F*. *prausnitzii* shows the highest potential for supporting requirements of a large number of community members (values range 0.036–0.313), from both bifidobacteria and lactobacilli. Several authors reported crossfeeding between (i) acetate-depending butyrate-producing colon bacteria like *F*. *prausnitzii*; (ii) lactate- and acetate-producing bacteria, like bifidobacteria [23–25]; and (iii) lactobacilli [24]. For example, Rios-Covian et al. [23] showed experimental evidence of crossfeeding between *F*. *prausnitzii*, which requires acetate for the oligofructose breakdown, and *Bifidobacterium adolescentis*, which produces acetate. *B*. *adolescentis* enhances its growth through consumption of carbohydrates, which are in turn released during oligofructose degradation by *F*. *prausnitzii*. These interactions are strain-specific and can be either a commensal beneficial relationship or dominated by competition [26].

*A*. *muciniphila* is the least competitive species within our microbial community with *metabolic competition* values ranging from 0.348 of *L*. *reuteri* to 0.644 of *L*. *rhamnosus*. Also *F*. *prausnitzii* shows values that characterize them as a non-competitive species. By contrast, the closely related species *L*. *casei* subsp*. casei* and *L*. *paracasei* subsp*. paracasei*, which belong to the same phylogenetic group [27], are the most competitive pair (0.947/0.968).

Within bifidobacteria genera, *B*. *adolescentis* and *Bifidobacterium longum* subsp. *longum* are the least competitive pair (0.703 and 0.811) while *Bifidobacterium pseudocatenulatum* and *B*. *catenulatum* are the most competitive pair (0.939 and 0.909) followed by *Bifidobacterium longum* subsp*. infantis* and *B*. *longum* subsp. *longum* (0.873 and 0.891). However, species pairs within the bifidobacteria genus are generally very competitive (mean competition index of 0.821).

Competition scores for lactobacilli range from 0.419 (*L*. *reuteri/L*. *acidophilus* pair) to 0.968 (*L*. *paracasei* subsp*. paracasei*/*L*. *casei* subsp. *casei* pair). The scarce competitiveness of the unique homo-fermentative *L*. *acidophilus* with other lactobacilli, among other factors, might be linked to its exclusive fermentation of hexoses to lactic acid via the Embden-Meyerhof pathway [28]. Indeed, by contrast, several facultative hetero-fermentative species (*Lactobacillus plantarum*, *L*. *rhamnosus*, *L*. *casei* subsp*. casei*, *L*. *paracasei* subsp*. paracasei*), which can ferment both hexoses and pentoses to lactic acid by the phosphogluconate pathway, in our study stand as high competitors (high competition scores), mainly when paired with *L*. *acidophilus*.

Considering our whole community, *A*. *muciniphila* and *F*. *prausnitzii* are the least competitive when paired with species of other genera, possibly due to their very distinct set of exogenous metabolites.

**Fig. 4:**
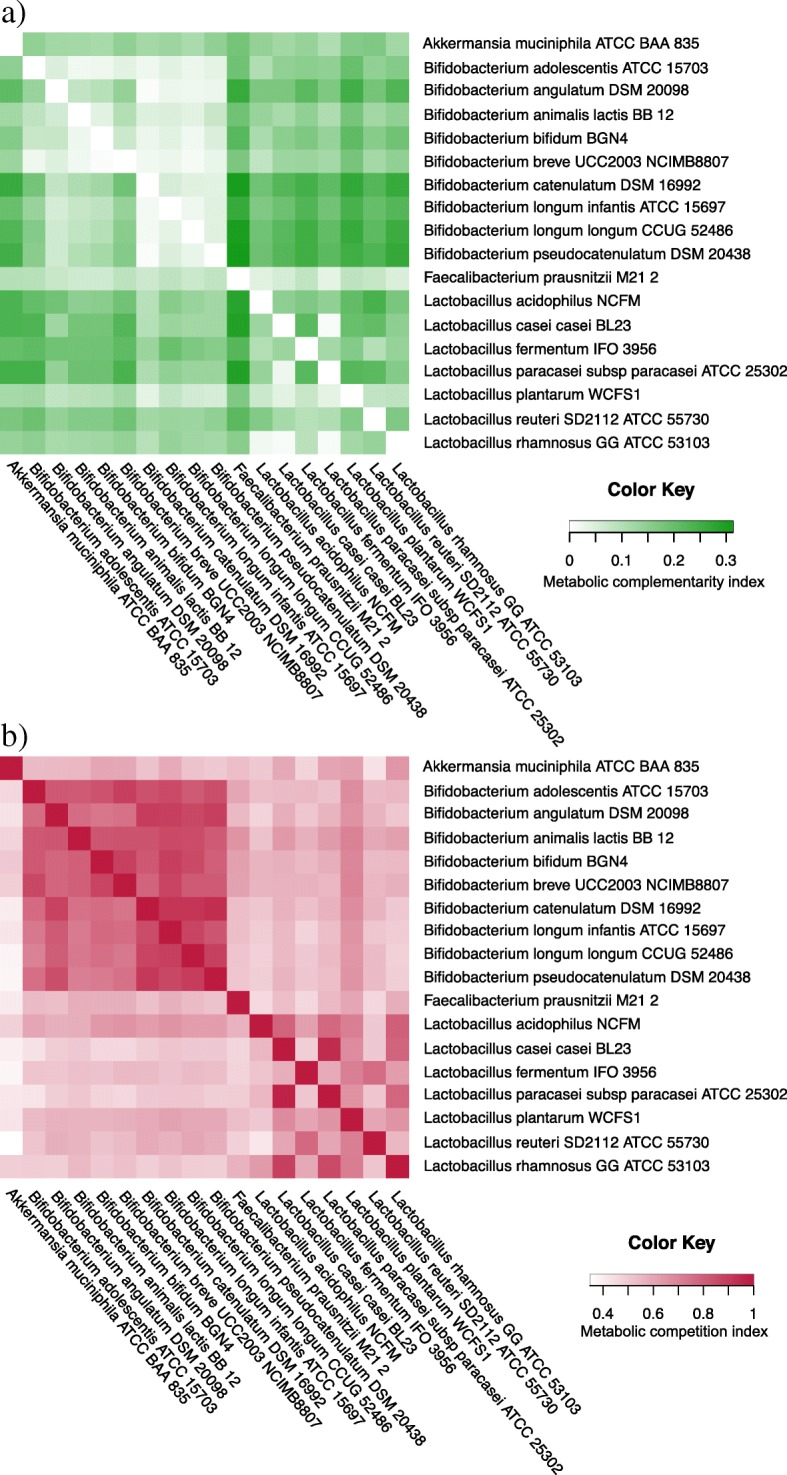
Microbial interaction matrix. **a** Metabolic complementarity index. For each pair, the complementarity color represents the ability of a microbe in a column of complementing the nutritional requirements of a species in a row. **b** Metabolic competition index. For each pair, the competition color represents the competition that a species in a column can exert on a species in a row

A graphical summary of the metabolic complementarity and competition indices is given in Fig. 4a and b, respectively while numeric data for support, complementarity, and competition is reported in Additional file 5.

### Sourcing exogenous feed metabolites (candidate prebiotics) from whole foods (question iv)

**Fig. 5:**
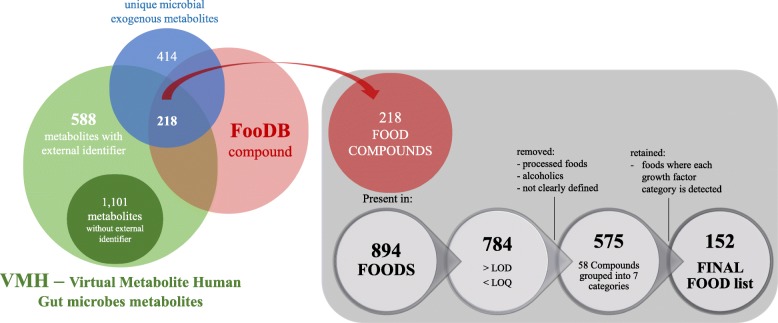
Flow diagram illustrating the process of food selection in FooDB, starting from microbial exogenous metabolites derived from VMH. Of the 632 exogenous metabolites from VMH, 218 corresponded to food compounds from FooDB, which were present in 894 foods. After selection for LOQ (limit of quantification) and removal of processed foods, alcohol, not clearly defined entries, and foods where at least one of the growth factor categories (amino acids, vitamins oligo, and monosaccharides) was not detected (LOD: limit of detection), the final foods selected were 152

Among the 632 identified exogenous metabolites, each needed by at least one of the bacteria in our probiotic community, there are 218 name-matched compounds in the food compound list of the Foods database (FooDB)—candidate prebiotics—in 894 whole foods (Fig. 5).

The VMH database includes internal metabolites (1101 out of a total of 1689 metabolites for the 773 gut microbes reported by Magnúsdóttir et al. [29]), which are not present in other databases: therefore, these metabolites do not have a match in FooDB. Moreover, not all metabolites might be present as compound in foods and for this reason only 218 over 632 metabolites from VMH were found in FooDB. Interestingly, around 60% of the candidate prebiotics are present in several common fruits and vegetables: specifically, 62.7% are present in potatoes, 61.0% in cucumbers, and 59.3% in spinaches, broccoli, and common pea. Minerals, such as potassium, sodium, magnesium, and zinc, are the most commonly found (between 95.4 and 92.3%), and also vitamins, such as pyridoxine and β-carotenes, are widely present in most of the foods (98.5 and 80.7%, respectively).

**Fig. 6:**
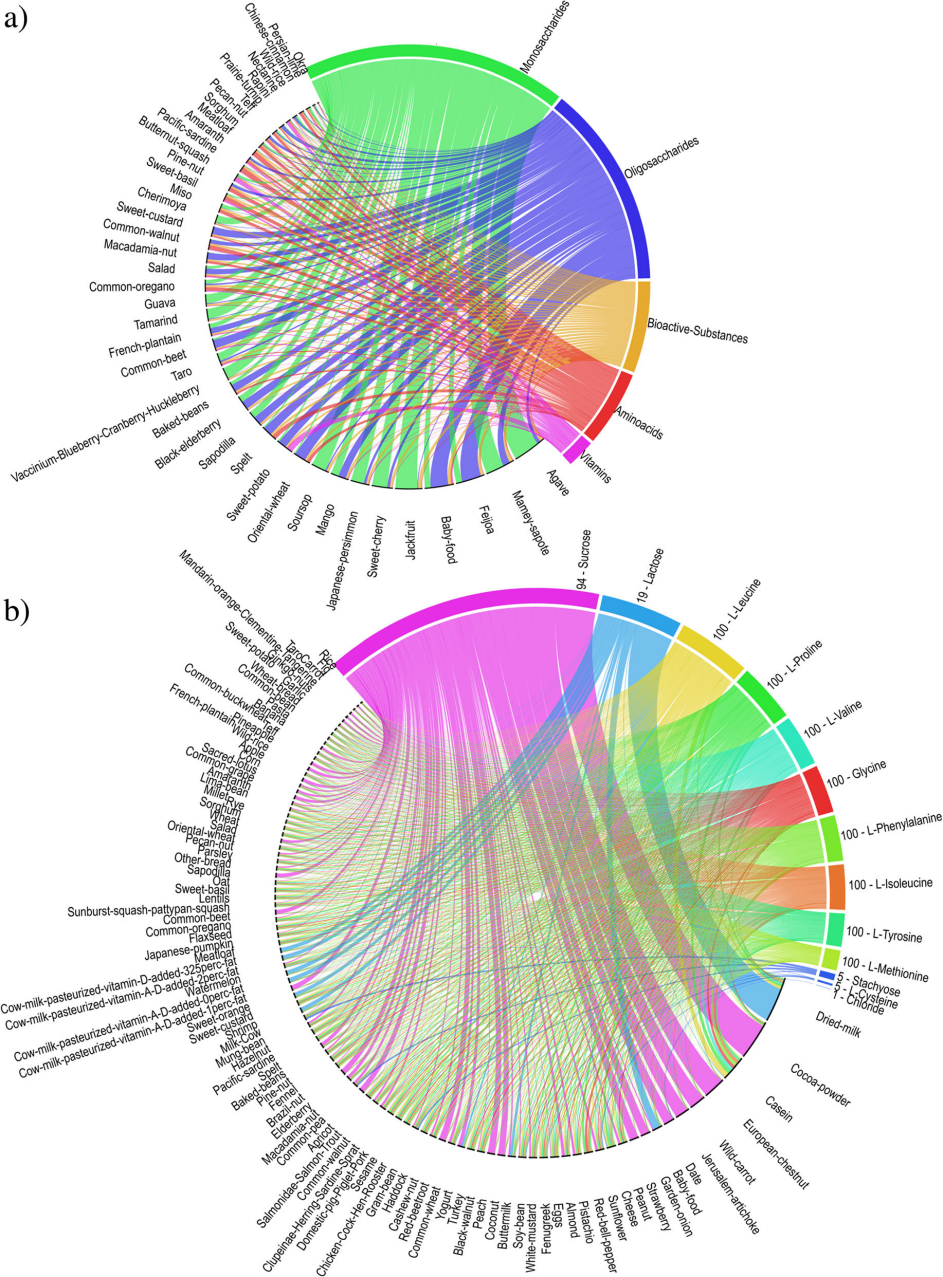
**a** Circos relations between metabolite categories (oligosaccharides, monosaccharides, amino acids, vitamins, nitrogenous compounds, and other bioactive substances) required by our selected microbial community and the FooDB-derived foods. Only foods which include all metabolite categories are shown. The thickness of the arches (colored band) indicates the mean concentration of the metabolite category in associated foods (1 mg/100 g). **b** Circos relations between all individual metabolites required by the selected microbial community and the FooDB-derived foods. Only foods which include more than nine (70%) of community-required metabolites are shown (100 foods in total). The number preceding the metabolite represents the number of foods that are linked to each category (e.g. 100 means that the metabolite was found in all the 100 identified foods). The length of the arches (colored band) indicates the mean concentration of the metabolite in associated foods (1 mg/100 g)

For further analysis, metabolites were then categorized into *oligosaccharides*, *monosaccharides*, *amino acids*, *vitamins*, *nitrogenous compounds*, *bioactive substances*, and *non-standard nutritive compounds* (see Additional file 3: Supplemental Information 1). For each compound category, the mean metabolite concentration was calculated. The relationship between metabolite categories and foods, after removing non-standard nutritive compounds (NSN) and removing foods with metabolites below the limit of quantification (LOQ), is shown in the circular visualization of Fig. 6a. Figure 6b shows the Circos between those exogenous metabolites that are shared among microbes in our community and could be found in FooDB and the associated foods (13 metabolites after removing NSN).

**Fig. 7:**
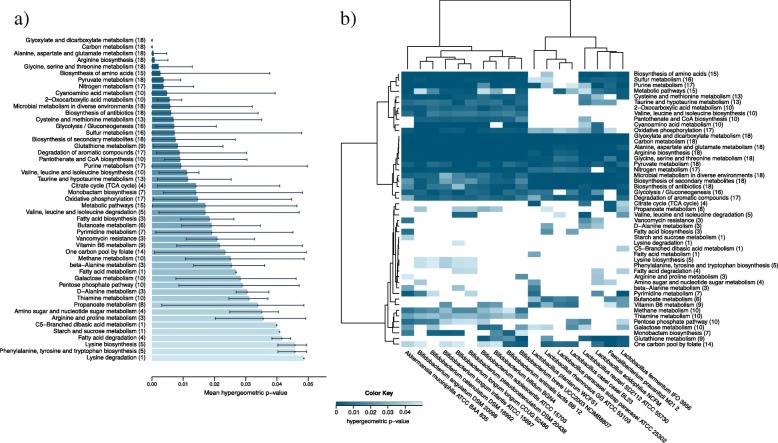
Metabolite pathway enrichment analysis performed with bStyle. **a** Histogram: *X*-axis shows, for each pathway, the mean of the hypergeometric *p* value measured for each microorganism; in addition, the number of microorganisms, in which the pathway is overrepresented is given in parenthesis. **b** Heatmap: hierarchical clustering of the metabolite pathway enrichment analysis. Color legend: white = pathway not detected in the microbe of interest; blue = statistical significance (higher blue intensity indicates higher statistical significance for the enriched pathway for the set of selected exogenous metabolites). The number of microorganisms, in which the pathway is overrepresented, is given in parentheses

A metabolite pathway enrichment analysis was performed for our probiotic community using bStyle [30]. Pathways were considered significant if a hypergeometric *p* value lower than 0.05 was obtained. The histogram in Fig. 7a and the heatmap in Fig. 7b show pathways whose average overrepresentation significance is less than 0.05 (full results for each microorganism are available in Additional file 6).

The highest enriched pathways are glyoxylate, dicarboxylate, and carbon metabolism (hypergeometric *p* value of 0.0000119 and 0.0000185, respectively) appearing in all members of the microbial community selected in our study. Seven additional pathways are also significantly enriched: alanine, aspartate, and glutamate metabolism (*p* = 0.00054); arginine biosynthesis (*p* = 0.00084); glycine, serine and threonine metabolism (*p* = 0.0021); pyruvate metabolism (*p* = 0.0037); microbial metabolism in diverse environments (*p* = 0.0058); biosynthesis of antibiotics (*p* = 0.0061); and biosynthesis of secondary metabolites (*p* = 0.0075). The metabolic pathways of carbohydrates, co-factors, vitamins, and amino acids are also overrepresented, even with lower, but still significant, *p* values. Our analysis also revealed four pathways that are overrepresented in only one species each, and these are fatty acid metabolism in *L*. *plantarum* (*p* = 0.0015), C5-branched dibasic acid metabolism in *Lactobacillus fermentum* (*p* = 0.0022), starch and sucrose metabolism in *A*. *muciniphila* (*p* = 0.0023), and lysine degradation in *B*. *longum* subsp. *longum* (*p* = 0.0027). The metabolic enrichment analysis confirmed the role of essential compounds (see Additional file 4) in vitamin B and amino acid metabolism as growth factors for the selected microbial community of 18 probiotics [31, 32].

The foods were then sorted according to their nutritive content (Additional file 7 and top 20 foods and their content in Table 3). At the top of the food list, there is “honey” that is much enriched in monosaccharides and bioactive substances, while “Red bell pepper” is characterized by high amounts of vitamins and amino acids.

**Table 3:** Top 20 foods sorted by decreasing content in vitamins, amino acids, oligosaccharides, monosaccharides, bioactive substances, and N-compounds and increasing content of NSN. For each food, the mean concentration of each metabolite group (mg/100 g) and their prebiotic food score are shown. The “Baby Food” category is considered as a “control” and appears in an italic font

Food	Vitamins	Amino acids	Oligosaccharides	Bioactive substances	N-compounds	Mono-saccharide	NSNs
Honey	0.38	7.92	426.39	1361.06	0.00	9352.92	0.00
Red bell pepper	543.45	1087.16	3.06	469.98	0.60	388.13	0.02
Eggs	118.84	845.94	115.04	309.86	0.00	23.27	0.00
Cheese	40.45	785.12	176.39	287.93	0.00	14.81	0.00
Turkey	104.03	745.09	16.83	261.86	0.00	29.38	0.00
Sweet potato	591.60	53.05	858.19	203.09	0.00	161.88	0.00
*Baby food*	*59.27*	*161.20*	*1867.93*	*345.37*	*0.00*	*619.90*	*0.00*
Cattle (beef, veal)	39.81	782.68	1.18	259.46	0.00	1.99	0.00
Chicken (cock, hen, rooster)	68.66	678.95	12.66	234.91	0.00	16.56	0.00
Spelt	0.41	449.78	956.67	268.16	0.00	135.00	0.00
Domestic pig (piglet, pork)	25.83	680.78	24.34	242.96	0.00	61.64	0.00
Feijoa	0.34	19.17	2056.67	322.34	0.00	658.75	0.00
Oriental wheat	4.83	326.31	1191.67	254.74	0.00	130.00	0.00
Papaya	24.04	17.22	1598.75	356.70	0.00	1197.50	0.00
Peanut	34.67	700.12	477.08	318.34	0.00	143.65	0.01
Sweet basil	181.21	416.94	1.67	215.28	0.00	125.00	0.00
Common walnut	57.81	435.32	405.00	202.80	0.00	21.25	0.00
Italian sweet red pepper	542.47	58.88	4.58	158.18	0.00	519.38	0.00
Pacific sardine	22.97	545.28	5.00	196.02	0.00	50.00	0.00
Mamey sapote	8.45	52.78	745.00	379.17	0.00	1958.75	0.00

## Discussion

From the whole body of sentences extracted by the natural language processing (NLP) pipeline, after a manual curation, we defined a final gut microbial sub-community composed of 18 bacteria (Table 2 and Additional file 3: Table S1) that should as a community have a beneficial effect on the infant health.

This prompted us to investigate cooperative and competitive interactions within the community and its assembly rules applying a reverse ecology approach. Pair-wise microbial interaction mainly depends on the environment, into which two considered species are placed, and the availability of nutrients for their growth [21]. In a given ecological model with limited nutrient supply, high complementarity and low competition between two microbial species can suggest potential syntrophy or niche complementarity [33], but not necessarily a coexistence. Niche complementarity may occur in different forms according to different relations of predation susceptibility and resource dependence—including different forms of the same chemical resource—in a community of coexisting bacterial species where no one predominates [34]. Moreover, in closely related species, which are expected to share specific metabolic traits that help adapt to a specific habitat, a “habitat filtering” effect may be observed and nutrients available from the environment rather than crossfeeding between bacteria shape their community [21]. Nevertheless, lactis acid bacteria can produce antimicrobial peptides, i.e., bacteriocines, which play a role in the competitive exclusion of pathogens [35], as well as interference competition among closely related species [36], helping producers to colonize and establish a niche in the environment [37]. In summary, complementarity and competition metrics could reveal the pressure of the habitat on the gut community assembly.

Competition and complementarity scores were calculated for each microbe-microbe pair in the community, and support scores were calculated for each microbe-human host pair. The low support scores (~ 0.54) observed between each microbial strain and the human host supports the premise that these bacteria are non-pathogenic for humans, because pathogens (specifically intracellular organisms) typically show high support values (> 0.7) [21]. Competition and complementarity were used to capture species interactions within the community; indeed, complementarity is a measure of potential syntrophy between two species, while competition index provides a proxy for niches overlap.

Reverse ecology analysis revealed generally low complementarity (< 0.32) and high competition scores (~ 0.97) for probiotic species pairs in our microbial community. We interpreted the results assuming the co-occurrence of these species in our ecology model. Low complementarity combined with high competition values observed for bacteria pairs belonging to the same genus suggest that these pairs are close relatives with similar nutrient needs, although the contribution of bacteriocines to modulate the microbial community could also be a major determinant. These nutrients are mainly acquired from the environment rather than via crossfeeding, and, hence, the bacteria may compete with each other, but do not necessarily exclude one another. Species pairs within the genus bifidobacteria are the most competitive and show the lowest potential for syntrophy; in particular, *B*. *longum* subsp. *infantis* is the most competitive. The highest competition scores were obtained for evolutionary closely related pairs. Among bifidobacteria, *B*. *pseudocatenulatum* exerts the highest competitive interaction when paired with *B*. *catenulatum*. Among lactobacilli, *L*. *paracasei* subsp*. paracasei* and *L*. *casei* subsp*. casei* are the most competitive pair. Interestingly, the least competitive pair among bifidobacteria, *B*. *longum* subsp*. longum* and *B*. *adolescentis*, consists of species belonging to two phylogenetically distinct bifidobacteria groups as evident by their distant positions on the recently reconstructed phylogenetic tree for the *Bifidobacterium* taxon [38].

*A*. *muciniphila* and *F. prausnitzii*, which have nutritional needs very different from the others and *L*. *acidophilus*, show low competition values. Hence, we can assume they do not compete with other species in our selected microbial community. Those species might be able to grow mutualistically with the other microbes because their nutritional needs could be met by crossfeeding [33]. While *A*. *muciniphila* is not affected by competitiveness from other microbes, *L*. *acidophilus* can encounter competition from facultative hetero-fermentative lactobacilli. The low competition and the relatively high complementarity value of *A*. *muciniphila* suggests its important metabolic role within our selected microbial community; a capable mucin degrader and butyrate producer, it is involved in crossfeeding supporting the growth of several other species. Mutualistic interactions might also occur between some lactobacilli and bifidobacteria species pairs, e.g., *B*. *catenulatum*, *B*. *longum* subsp. *longum*, *B. longum* subsp. *infantis*, and *B. pseudocatenulatum*. On the other hand, *B*. *breve* and *B*. *adolescentis*, which show comparatively high competition values and are not much involved in potential syntrophic relations (low complementarity), might exert competitive behavior in the community*.* From the results, we might suppose the possible competition between *F*. *prausnitzii*, with *Bifidobacterium animalis* subsp. *lactis* and with *B*. *bifidum.* In a nutshell, our reverse ecology analysis provided information on potential [39] competitions and/or complementations between selected species in our community. These potential outcomes are strain-dependent as revealed by Moens and co-authors [26]. Performing co-culture fermentations, they found a strain-dependent interaction between bifidobacteria strains and *F*. *prausnitzii*, which can be either commensal or competitive depending on the inulin-type fructanse degradation capacity of the former*.*

The most over-represented metabolic pathways covered by these compounds are classified into the Kyoto Encyclopedia of Genes and Genomes (KEGG) [40] classes of carbohydrate and amino acid metabolism. Among carbohydrates, the galactose metabolism and the pentose phosphate pathway are enriched by seeds from 10 out of 18 species of the community. The metabolic pathway enrichment analysis showed d-glucose, sucrose, lactose, d-sorbitol, galactitol, d-mannose, *N*-Acetyl-d-galactosamine, alpha-d-galactose, and myo-inositol as associated to the galactose metabolism. Moreover, the fundamental role played by microbial growth factors found in the vitamin B and amino acid metabolism has been underlined.

The aim of introducing prebiotics into the diet is to stimulate growth of specific indigenous, health-beneficial gut bacteria, i.e., probiotics. Oligosaccharides are established prebiotic substrates because they are carbohydrates non-digestible for the human host and can therefore reach the colon, where they can be selectively fermented by indigenous beneficial bacteria [41]. Other substrates used as prebiotics include fibers, cellulose, hemicellulose, pectins, gums, β-glucans, inulin, fructose-oligosaccharides, and galacto-oligosaccharides [42]. In addition to these prebiotics, specific growth factors are also required by gut microbes, such as B vitamins and amino acids, essential for both bifidobacteria [31] and lactobacilli [32]. The metabolic pathway enrichment analysis shows that most of the exogenous metabolites identified for lactobacilli and bifidobacteria belong to vitamin B and amino acid pathways.

In order to inform the design of a prebiotic complementary infant feeding, the metabolites that are essential for at least one member of the selected microbial community (i.e., a total of 632 metabolites) were sourced into the FooDB. All 218 matching compounds were associated with a final number of 575 foods. The foods were sorted (Additional file 7) for facilitating the selection of the most promising food groups suitable to nourish and support the growth (prebiotic food) of these selected bacterial species (probiotics). We propose the top 20 foods (Table 3) that include vegetables and fruits, which are natural resources of prebiotic compounds, and moreover, most of them are normally recommended as complementary foods which can provide infants a large proportion of micronutrients such as iron, zinc, phosphorus, magnesium, calcium, and vitamin B6 [43]. Among the foods showing the highest amounts of vitamins and amino acids, sweet potato (Kumara) appears as a practical choice for the formulation of infant complementary foods. Kumara powder is therefore proposed for the pilot clinical trials on infants aged 6–12 months, where each subject will receive a fixed quantity that could be mixed with the infant’s complementary foods.

This proof-of-concept of a health benefit-driven, public domain mining reverse metabolic approach to identifying candidate pro- and prebiotics and matching food sources comes with some limitations: For example, we initially encountered a number of false positives from the “text mining” due to the complexity of the search criteria; we have largely overcome this limitation by optimizing our dictionaries and adopting additional filters to exclude non-relevant articles. For our proof-of-concept, we deliberately selected a probiotic microbial community for gut ecology analysis. However, the infant gut harbors many more microbes apart from those selected by us and, hence, the interactions predicted by NetCooperate will be much more complex and may even differ for our selected community when embedded into a larger microbial context. Our exogenous metabolite (prebiotic) identification was targeted towards the strains that are available in VMH (which again depends mainly on literature for metabolite reconstruction). From the literature, it is evident that the prebiotic requirement is strain-specific. Hence, the full potential of growth-promoting function of this complementary feeding may not be realized fully in clinical studies conducted with a heterogeneous population. Moreover, we encountered some nomenclature limitations while mapping metabolites between VMH and FooDB databases, that calls for an increased effort in unifying the identifiers and give researchers full instruments for the inter-resources data integration. However, to the best of our knowledge, this is the first in silico study to identify prebiotics for the growth of potential probiotics that beneficially impact on the immune system development in infants during weaning.

## Conclusion

In conclusion, deploying complex public domain mining and developing an in silico reverse metabolic approach, we have (i) constructed probiotic-prebiotic-food correlation matrices that can guide targeted feeding of immune health-beneficial microbes by weaning food in infants; (ii) analyzed competition and synergy for (prebiotic) nutrients between microbes; and (iii) translated this information into designing an experimental complementary feed for infants enrolled in a pilot clinical trial (http://www.nourishtoflourish.auckland.ac.nz/). The presented, benefit-oriented microbiome research strategy is in our opinion nutritionally more actionable than large-scale descriptive studies in humans and has potential to be more translational than microbiomics in rodent models, be they mouse strains, genetically engineered models, or gnotobiotic mice seeded with a limited set of microbes. Our reverse metabolic pipeline aimed at leveraging in silico-generated data into clinical relevance. We have tried to extend from “classical” to molecular nutrition, with the aim of identifying nutrients, bacteria, and mechanisms that point towards crossfeeding to improve immune health in infants around weaning. Our “seeding through feeding” approach differs fundamentally from classical prebiotic candidate testing (feeding baby to assert microbial changes and clinical outcomes). We deliver here the proof-of-concept showing that the in silico mining indeed yields new pro- and prebiotics related to healthy infant immunity but also “positive controls,” i.e., bacteria we expect to find, such as bifidobacteria and lactobacilli. Overall, we present an innovative, translational systems biology-based methodology to inform feeding the infant gut with the *optimum* prebiotics to facilitate the growth of beneficial gut bacteria that help the maturation and development of immune system in human infants.

## Methods

To identify exogenous metabolites required by gut microorganisms potentially capable of enhancing the immune system against infant infections and to detect those metabolites in foods/food components, we developed an integrated pipeline based on literature searching, text mining, reverse metabolic analysis, and food database exploration.

### Identification of immune-protective bacteria (question i)

The workflow for the identification of immune-protective bacteria is shown in Fig. 1. As a preliminary step, all context-relevant concepts and keywords were identified. To prioritize and focus on the research aims, the initial concepts were organized into four research topics: (a) infant gut microbiome, (b) infant nutrition and microbiome metabolism, (c) beneficial bacteria supporting immune system development in infants, and (d) beneficial bacteria preventing infections in infants. For each topic, a specific literature query was formulated (see Additional file 3: Supplemental Information 3 for explanations). To enhance the specificity of the queries, keywords specifically excluding non-human/non-infant data were taken into consideration. However, some of those keywords, especially “rat/mice model” and “in vitro” study, occur frequently in literature to provide context for the article. Hence, those excluding terms were retained in the queries and used downstream for further manual filtering of the results, therefore yielding both fewer false-positive and false-negative articles.

The literature was queried (31 October 2017) for each of the four research topics to retrieve eligible MEDLINE abstracts and/or full texts from the PMC Open Access Subset and PMC Author Manuscript Collection repositories in PubMed Central. The obtained collection of documents, representing the relevant *literature corpus* (3673 PubMed IDs), was annotated for key biological entities, (genes, chemicals, species, DNA mutations, SNPs, protein mutations, and diseases) using state-of-the-art biomedical annotation tools: DNorm [44], tmVar [45], tmChem [46], and GNormPlus [47]. The tools are the same as utilized in the Pubtator database, which was deployed for abstract annotations [48], as it allows the downloading of the data (revision date: 26 June 2017).

All biomedical annotations were retrieved from the relevant literature corpus of abstracts and full texts and were manually inspected to improve both understanding and extent of the potential biological concepts involved in the weaning period. Pertinent annotations and entity lists of terms were therefore collected in three dictionaries: (i) immunity-infant related, (ii) infant infection-disease related, and (iii) chemical related (Additional file 3: Supplemental Information 4). Moreover, a fourth microorganism dictionary was included with names of human gut microorganisms. To build this dictionary, a complete list of microbes at genus level colonizing the human GI tract was used from the Human Microbiome Project catalogue [49]. Two further lactic acid bacteria genera, *Oenococcus* and *Lactococcus*, were included. The former, *Oenococcus*, which is commonly used in wine fermentation, was not reported as a gut commensal in the HMP catalogue, but appears in the Metagenomics of the Human Intestinal Tract (MetaHit) catalogue, and it is present in 13.5% of subjects of the Danish and Spanish cohort (249 individuals) [50]. The latter, *Lactococcus*, is a genus dominating in the baby gut microbiota [51]. Furthermore, the complete list of microbes at *species* level was downloaded from the NCBI taxonomy [52] and manually curated for non-pathogenic species relevant to human gut alone. Opportunistic pathogens and other non-relevant bacterial species (those not described at genus level in the Human Microbiome Project) were excluded from the final list.

The four dictionaries were combined with the literature corpus and submitted to natural language processing (NLP) methods to extract relevant information for the four research topics of the project. The CoreNLP API [53] was used to parse the scientific text in the corpus, and an analytical pipeline was developed to identify the linguistic relations between the concepts found in our dictionaries. This is the most important step: all abstracts and full texts of the literature corpus were automatically annotated and analyzed to identify relevant linguistic relationships between at least one pair of terms from each pair of dictionaries. Co-mention between at least one term related to more than two dictionaries, for all combinations, was also obtained. Table 1 contains the combinations of the four dictionaries used for NLP relationship and co-mention identification. Out of 3673 papers in our literature corpus of abstracts and full texts, the NLP pipeline extracted 348 unique relevant sentences for relations among two dictionaries and 129 for co-mentions among three or four dictionaries (an excerpt is shown in Additional file 3: Table S1). These were manually curated to ensure coherence, exclude false positives, and identify relevant bacteria at species/strain level, wherever possible. The latter step was carried out by exploiting the manually revised list of human gut microorganisms containing only non-pathogenic and, putative, probiotic bacteria. Results from text mining were filtered to exclude articles published before 2000, sentences from title journal section and from full texts containing unrelated terms (e.g., animals except mice…), “pathogen” or “not-relevant” species at genera level (e.g., species level). The role of the identified microorganisms in supporting the development of the immune system during early life was manually evaluated to define a set of putative probiotic bacteria for further analysis. The evidence on beneficial effects of those microbes was mainly obtained from clinical studies, with some relevant articles on in vitro effects being included in the manual curation process. The full list of sentences identified with text mining is available in Additional files 1 and 2.

### Identification of exogenous metabolites for selected microorganisms (question ii)

Exogenous metabolites required by beneficial microbes (putative probiotics) were identified by reverse metabolic analysis of their metabolic models. The metabolic models were those published in the Virtual Metabolic Human [54] database, which contains the in silico metabolic reconstructions of microbes commonly found in the gut [29]. Those are automatically reconstructed using ModelSeed [55] starting from the two aerobic bacteria *Bacillus subtilis* and *Escherichia coli* to evaluate stoichiometric coefficients for all gram-positive and gram-negative bacteria respectively and may thus represents a bias for anaerobic metabolism. A Perl script was implemented to transform these metabolic models, which are distributed in the Systems Biology Markup Language (SBML) format, into text-based metabolic networks best suited for the downstream analyses. The resulting text-based models are directed graphs, represented with the nodes corresponding to compounds and the edges corresponding to reactions linking reagents to products. Furthermore, the tool harmonizes in a detailed tabular representation all metabolic reactions of each organism, including enzymes and name identifiers from the universal metabolite names and CHEBI identifiers converted using the BiGG Models database [56]. To infer the nutritional profile of each beneficial microbe, the metabolic topologies so far obtained were analyzed with NetSeed [57], a tool to identify the minimal subset of nodes (in this case, compounds) that cannot be synthesized from other compounds in the network and therefore need to be acquired *exogenously* (see Fig. 3).

### Cooperative metabolic interactions between bacteria and between host and bacteria (question iii)

To deepen the insights into potential ecological microbial interactions between species in our selected community composed of selected candidate probiotics from the text mining analysis, a reverse ecology analysis was applied to predict the ecological structure of this community. For determining host-microbe and microbe-microbe cooperative and competitive potential in a pair-wise manner, NetCooperate [21] and the R package RevEcoR version 0.99.3 [58], in R version 3.4.1, were used. Three reverse ecology measures for species interactions were calculated.

The *biosynthetic support score* provides a measure for the capacity of a potential host to meet the metabolic requirements of a microbe and can be also regarded as a *cooperation* index [59]. The support represents the fraction of exogenous metabolites of the bacterium, i.e., the nutrients or precursors that cannot be produced by the bacterium itself, that are found in the metabolic interaction network of the host. Its value ranges from 0, no cooperation, to 1, full cooperation. While parasites “egoistically” exploit the host’s metabolism, commensal bacteria have a mutualistic relationship with the host, i.e., they both benefit and contribute. High support scores (> 0.75) are reported for parasites whereas low scores (< 0.75) are reported for commensals [21].

The *metabolic complementarity index* between two species measures the ratio (range 0–1) of exogenously acquired compounds in one species that are found in the metabolic network, although not as its feed, of the other species [33]. As such compounds are used by both species, niche complementarity and syntrophy are likely to happen and, typically, high complementary species are able to coexist in contrast to low complementary ones that do rather not co-occur [21].

The *metabolic competition index* of two species is the fraction of compounds exogenously required by both species and is therefore a measure of potential nutritional competition [33] with values ranging from 0 (no competition) to 1 (high competition).

### Sourcing exogenous metabolites from whole foods (question iv)

Exogenous compounds, which may inform dietary-based interventions, were used to interrogate the Canadian Foods database (FooDB), a resource of whole foods and food components, as well as their chemistry and biology. The exact chemical name of the metabolites from VMH was sourced into the compound database (1689 metabolites for the 773 gut microbes by Magnúsdóttir et al. [29]), and the identified compounds were linked to foods. We encountered a limitation in matching the nomenclatures used in VMH as it provides an extensive number of metabolites whose names, to the best of the authors’ knowledge, cannot be retrieved elsewhere (see the “Results” section for details). The list was manually filtered to exclude processed foods, alcohol, and those foods that do not contain clearly defined products (belonging to “Other …” categories, see Additional file 3: Supplemental Information 5). “Baby foods” category (see Additional file 3: Supplemental Information 2, for description) was selected as positive control for this study.

A metabolic enrichment set analysis was performed using the identified exogenous metabolites for which VMH reported a KEGG identifier. The hypergeometric distribution is used to evaluate how likely specific KEGG-available exogenously identified chemical compounds are associated to microbial-specific pathways and help identify those biological processes and pathways that best explain the meaning of the exogenous compounds. All analyses were performed using the graphical environment bStyle [30], which automated and simplified the entire integration and analytical process (Additional file 3: Figure S1). bStyle also proved to be much faster than analogous R implementations. All selected strains were available in the KEGG database [40], except *F*. *prausnitzii* M21 2 that was replaced by *F*. *prausnitzii* SL3/3 as it belongs to the same phylogroup [60] and *B. longum* subsp. *infantis* CCUG 52486 that was replaced by the closely related *B*. *longum* subsp. *longum* strain BBMN68 [61]. Only pathways in the “metabolism” KEGG class, which includes biochemical transformations—essential for growth, reproduction, maintenance of physiological structures, and response to environmental changes—were considered in bStyle. A hypergeometric *p* value lower than 0.05 was defined as the threshold for considering significant the enriched pathways.

To better classify the foods for further selection and trial in infant weaning studies, metabolites were grouped in seven categories: oligosaccharides, monosaccharides, vitamins, amino acids, other bioactive substances, organic nitrogen compounds, and non-standard nutritive compounds (NSN; see Additional file 3: Supplemental Information 1). The NSN category includes trace elements such as nickel, cadmium, and other heavy metals, plus compounds like acetaldehyde, ethanol. While some of these trace elements are co-factors, they exhibit very narrow concentration ranges for bioactivity, above which they can be or are toxic. Acetaldehyde and ethanol are common small organic compounds in human metabolism but not regarded as nutrients. Furthermore, those substances have been associated to health complications [62, 63]. This is why we termed this category as NSN.

The value of metabolite categories was computed, for each food, as the mean concentration (in mg/100 g) of their compounds normalized to the range [0–1]. The sum of the metabolic categories provides a nutritional-oriented ranking for the foods, and to prioritize foods rich in essential growth factors (vitamins and amino acids) that are usually found in very low concentrations, the weights 10, 8, 6, 4, 2, 1, and − 100 were used respectively for vitamins, amino acids, oligosaccharides, bioactive substances, N-compounds, monosaccharides, and NSNs.

Based on this sorting, the most promising whole-food source containing several of the identified candidate prebiotics can be chosen to be incorporated in an experimental complementary feeding for infants to support and enhance the growth of immune-protective beneficial microbes. This experimental weaning food will be administered in a pilot clinical trial to demonstrate the role of prebiotic complementary feeding in modulating the infant gut microbial composition and abundance (http://www.nourishtoflourish.auckland.ac.nz/).

## Additional files

Additional file 1: Relevant relationship for PubMed IDs. (XLSX 225 kb)

Additional file 2: Relevant co-mention for PubMed IDs. (XLSX 109 kb)

Additional file 3: **Table S1.** Example of relevant sentences from the pipeline for selected microbes. Supplemental Information 1 List of metabolites in each metabolite category. Supplemental Information 2 Description and motivation for “Baby Food” category. Supplemental Information 3 Literature queries for each topic. Supplemental Information 4 List of terms in each dictionaries. Supplemental Information 5 List of manually filtered foods, which excludes processed foods, alcohol and those foods that do not contain clearly defined products. Figure S1 Metabolic enrichment set analysis description. (PDF 1050 kb)

Additional file 4: Nutrient profile of each microbes in the community in exam as resulted from NetSeed. (XLSX 59 kb)

Additional file 5: Data for support, complementarity and competition. (XLSX 72 kb)

Additional file 6: Metabolite pathway enrichment analysis results for each selected microbes. (XLSX 108 kb)

Additional file 7: Foods sorted according to their nutritive content. (XLSX 27 kb)

## Abbreviations

CHEBI: Chemical Entities of Biological Interest
GI: Gastrointestinal
HMP: Human Microbiome Project
KEGG: Kyoto Encyclopedia of Genes and Genomes
LOD: Limit of detection
LOQ: Limit of quantification
MetaHit: Metagenomics of the Human Intestinal Tract
NLP: Natural language processing
SBML: Systems Biology Markup Language
VMH: Virtual Metabolic Human
