# Supplementary material for: A reverse metabolic approach to weaning: in silico identification of immune-beneficial infant gut bacteria, mining their metabolism for prebiotic feeds and sourcing these feeds in the natural product space
Source: Microbiome. 2018 Sep 21;6:171. doi: 10.1186/s40168-018-0545-x (PMC6151060; doi:10.1186/s40168-018-0545-x)
Supplement: Supplementary file 3 — Table S1. Example of relevant sentences from the pipeline for selected microbes. Supplemental Information 1 List of metabolites in each metabolite category. Supplemental Information 2 Description and motivation for “Baby Food” category. Supplemental Information 3 Literature queries for each topic. Supplemental Information 4 List of terms in each dictionaries. Supplemental Information 5 List of manually filtered foods, which excludes processed foods, alcohol and those foods that do not contain clearly defined products. Figure S1 Metabolic enrichment set analysis description. (PDF 1050 kb) [file 40168_2018_545_MOESM3_ESM.pdf]

Additional File 3

A reverse metabolic approach to weaning: *In silico* identification of immune-beneficial infant gut bacteria, mining their metabolism for prebiotic feeds and sourcing these feeds in the natural product space

Samanta Michelini[1], Biju Balakrishnan[2], Silvia Parolo[1], Alice Matone[1], Jane Mullaney[3], Wayne Young[3], Oliver Gasser[4], Corrado Priami[1,5], Rosario Lombardo[1*], and Martin Kussmann[2,6]

*** Correspondence:** Rosario Lombardo: lombardo@cosbi.eu

Abstract

Background: Weaning is a period of marked physiological change. The introduction of solid foods and the changes in milk consumption are accompanied by significant gastrointestinal, immune, developmental and microbial adaptations. Defining a reduced number of infections as the desired health benefit for infants around weaning, we identified *in silico* (i.e. by advanced public domain mining) infant gut microbes as potential deliverers of this benefit. We then investigated the requirements of these bacteria for exogenous metabolites as potential prebiotic feeds that were subsequently searched for in the natural product space.

Results: Using public domain literature mining and an *in silico* reverse metabolic approach we constructed probiotic-prebiotic-food associations, which can guide targeted feeding of immune health-beneficial microbes by weaning food, analyzed competition and synergy for (prebiotic) nutrients between selected microbes; and translated this information into designing an experimental complementary feed for infants enrolled in a pilot clinical trial (http://www.nourishtoflourish.auckland.ac.nz/)

Conclusions: In this study, we applied a benefit-oriented microbiome research strategy for enhanced early-life immune health. We extended from 'classical' to molecular nutrition aiming to identify nutrients, bacteria and mechanisms that point towards targeted feeding to improve immune health in infants around weaning. Here we present the systems biology-based approach we used to inform us on the most promising prebiotic combinations known to support growth of beneficial gut bacteria ('probiotics') in the infant gut, thereby favorably promoting development of the immune system.

Keywords: infant gut microbiome, prebiotic, probiotic, reverse ecology, infection, knowledge extraction, complementary feeding, systems biology

Supplemental Table 1

Example sentences, associated PubMed IDs and selected bacterial species obtained through text mining and full text analysis by our devised pipeline.

Candidate species	PubMed ID	Sentence	Notes
***Bifidobacterium adolescentis* ATCC 15703**	28056921	These results indicated that *B. adolescentis* protects premature rats from intestinal injury from hypoxia and cold stress, the mechanism of which might be by reducing the expression of TLR4 and increasing the expression of TOLLIP and SIGIRR. Although the pathogenesis of NEC is not entirely known [1], the theory that inappropriate microbial colonization in the intestine induces the immature intestine to mount an excessive inflammatory response that results in tissue injury and necrosis is a consideration [1].	mice
***Bifidobacterium animalis* subsp. *lactis* BB 12**	28027638	The present secondary analysis in a low-caries population evaluated the effect of early administration of *Bifidobacterium animalis* subsp. *lactis* BB-12	
***Bifidobacterium bifidum* BGN4**	28245626	*Bifidobacterium bifidum* OLB6378 (OLB6378), which belongs to the *Bifidobacterium* genus, could be a strong probiotic candidate that is capable of enhancing infants overall humoral immunity.	
	27649150	The various bio-functional effects and potential for industrial application of *B. bifidum* BGN4 has been characterized and proven by in vitro (i.e., phytochemical bio-catalysis, cell adhesion and anti-carcinogenic effects on cell lines, and immunomodulatory effects on immune cells), in vivo (i.e., suppressed allergic responses in mouse model and anti-inflammatory bowel disease), and clinical studies (eczema in infants and adults with irritable bowel syndrome).	
***Bifidobacterium breve* UCC2003 NCIMB8807**	26134988	TNF-α levels were significantly decreased in the first group after receiving *B. breve* for 3 months.	
***Bifidobacterium longum* subsp. *infantis* ATCC 15697**	22114588	Confirming and extending results of the first study, treatment with *B. infantis* (10^8 cfu/day) was associated with marked improvements such as lessening of (1) bloating/distention, (2) abdominal pain/discomfort, (3) passage of gas, (4) straining with defecation, and (5) sense of incomplete evacuation.	
***Bifidobacterium longum* subsp. *longum* CCUG 52486**	26985232	*B. longum* subsp. *infantis* CCUG 52486, combined with a prebiotic, gluco-oligosaccharide (*B. longum* + Gl-OS), on the response to seasonal influenza vaccination in young and older subjects in a double-blind, randomized controlled trial, taking into account the influence of immunosenescence markers at baseline.	
***Bifidobacterium pseudocatenulatum* DSM 20438**	28512033	The administration of *B. pseudocatenulatum* CECT 7765 to MS animals also reversed intestinal dysbiosis affecting the proportions of ten Operational Taxonomic Units (OTUs) at P21, which could partly explain the restoration of immune, neuroendocrine and behavioral alterations caused by stress in early and later life.	mice
***Lactobacillus acidophilus* NCFM**	28813007	The results of the present study demonstrated that a probiotic formula containing *Bifidobacterium longum* BORI and *Lactobacillus acidophilus* AD031 reduced the duration of rotavirus diarrhea in young Korean children.	

	10575148	Reduced incidence of necrotizing enterocolitis associated with enteral administration of *Lactobacillus acidophilus* and *Bifidobacterium infantis* to neonates in an intensive care unit.
***Lactobacillus casei* subsp. *casei* BL23**	22672413	Finally, Penders et al. [38] showed a decreased risk of atopic dermatitis in children colonized by *L. paracasei* , a member of the *L. casei* et rel.
	19946408	However, the number of rhinitis episodes was lower in the probiotic group leading the authors to conclude that *Lactobacillus casei* may benefit children with allergic rhinitis but not asthmatic children. One randomized placebo-controlled crossover study examined the effect of yogurt containing *S. thermophilus* and *Lactobacillus bulgaricus* when given with or without *Lactobacillus acidophilus* to adolescents and adults with asthma who were sensitized to inhalant allergens.
***Lactobacillus fermentum* IFO 3956**	23197978	Other studies demonstrated a significant reduction of severity scoring of atopic dermatitis index using *Lactobacillus fermentum* VRI-033 PCC 1 x 10^9 CFU twice a day during 8 weeks [113] and *Lactobacillus* GG in infants suspected cow's milk allergy IgE-sensitized [114].
***Lactobacillus paracasei* subsp. *paracasei* ATCC 25302**	28654019	Significant changes in innate and acquired immunity biomarkers were observed only in subjects in group A. Conclusions: Dietary supplementation with cow's skim milk fermented with *L. paracasei* CBA L74 is an efficient strategy in preventing CIDs in children.
	23282383	In children, the use of fermented milk fortified with *L. paracasei* LP33 has been proposed for the treatment of perennial allergic rhinitis (PAR) and has achieved significant reduction in pediatric rhinitis quality of life [104].
***Lactobacillus plantarum* WCFS1**	23462584	In the second child (11-year-old) who had symptoms of abdominal distension, watery and intermittent bloody stools and arthritis, treatment with *Lactobacillus plantarum* 299V (10^{10} CFU daily) facilitated discontinuation of antibiotics, PN, as well as medication for arthritis.
***Lactobacillus reuteri* SD2112 ATCC 55730**	28858247	The anti-inflammatory cytokine interleukin (IL)-10, was increased in newborns receiving *L. reuteri* DSM 17938.
	28858247	Also, fecal calprotectin, a well-known marker of gut inflammation, was reduced in the infants supplemented with *L. reuteri* DSM 17938.
	25245226	Prophylactic *L. reuteri* supplementation is as effective as nystatin, and more effective in reducing the incidence of proven sepsis in addition to its favorable effect on feeding intolerance.
	19946408	The incidence of IgE-associated dermatitis, rather than other types of atopic dermatitis, was decreased after the oral consumption of probiotics, namely *L. reuteri* or a mixture of four probiotic bacteria and prebiotics [31,32].
***Lactobacillus rhamnosus* GG ATCC 53103**	26271359	Likewise, in formula-fed infants, IgA immune response was reported to be enhanced both by *B. lactis* and *L. rhamnosus* [16].

	24860569	*L. rhamnosus* CRL1506 significantly modulated the production of type I IFN and IL-6 in the response to poly(I:C) or RSV challenges.
	24860569	Therefore, *L. rhamnosus* CRL1506, through the stimulation of anti-viral defenses in epithelial cells, could play a significant role in the improvement of innate and specific immune responses against respiratory viral infections (83).
Akkermansia muciniphila **ATCC BAA 835**	27129739	A protective or anti-inflammatory role in the intestinal mucosa has been proposed for this bacterium and reduced abundance of *Akkermansia* in humans has been previously shown in obese individuals and people with ulcerative colitis and Crohn's disease, underscoring the importance of this bacterium for gut health 52.
Faecalibacterium prausnitzii **M21 2**	27286235	This finding of decreased *F. prausnitzii* was consistent with previous findings in both pediatric and adult IBD (35), and is consistent with observations this organism may have anti-inflammatory properties through direct effects on cytokine production (36), as well as increased production of short-chain fatty acids such as butyrate (37).

Supplemental Information 1

Metabolites for each category:

1. **oligosaccharides** (*oligo*): sucrose, lactose, maltose, stachyose, raffinose, dextrin
2. **monosaccharides** (*mono*): D-fructose, D-glucose, D-galactose, D-glucitol, L-arabinose, D-mannitol, D-mannose,aArbutin
3. **vitamins** (*vit*): biotin, retinol, myo-inositol, pyridoxine, choline, nicotinamide
4. **amino acids** (*aa*): L-leucine, L-alanine, L-proline, glycine, L-valine, L-tryptophan, L-serine, L-isoleucine, L-phenylalanine, L-threonine, L-tyrosine, L-histidine, L-methionine, L-asparagine, L-cysteine, L-citrulline, ornithine, NG,NG-dimethyl-L-arginine
5. **bioactive substances** (*bioactive*): indole, allantoin, L-cystathionine, chloride, nitrate, magnesium, sulfur, sodium, potassium, zinc
6. **organic nitrogen compounds** (*N-comp*): putrescine, spermidine, adenine, urea, taurine
7. **non-standard nutritive compounds** (*nsn*): nickel, cadmium, lead, ethanol, acetaldehyde

Supplemental Information 2

"Baby food" was not excluded and selected for representing the positive control. It is a category into the FooDB that includes "Baby food, infant formula, high iron content, powder" and "Baby food, fortified cereal bar, fruit filling" and is defined as "*Baby food is any soft, easily consumed food, other than breastmilk or infant formula, that is made specifically for infants, roughly between the ages of four to six months to 2 years. The food comes in multiple varieties and tastes may be table food that the rest of the family is eating that has been mashed or otherwise broken down or can be purchased prepared from manufacturers.*"

Supplemental Information 3

Queries for each research topic.

Queries were constructed intersecting (operator AND) groups of concepts and their synonyms (used as alternatives, operator OR) related to specific areas (ex. microbiome, infants, infant nutrition, microbiota metabolism, immune system, infections, …) for retrieving specific PubMed IDs connected to the defined research topics. Few putative probiotic genera were used in the search query for validation purposes although they were not required to appear in the results (OR operator). The operator NOT was used to exclude animals, and *in vivo/in vitro* and animal model studies. Quotes were used to search for exact words, and where they are missing, the term is automatically searched also for its orthographic variants. Research topics queries were performed on title and abstracts to obtain the highest recall and collect most of potentially relevant PubMed IDs for our study. Further mining analysis were performed on available full-texts (or abstracts if not) from this refined collection of IDs.

(a) infant gut microbiome: groups of keywords were collected and intersected to select abstracts about structure and functions of the gut microbiome in infants in their first 1000-day of life.

(title,abstract:((((microbe OR microbial OR bacterial OR gut OR microbiome OR "microbial genome" OR symbiotic OR microbiota OR microflora OR "Gastrointestinal microbiome" OR metanalysis OR "meta-analysis" OR metagenome OR "meta-genome" OR archea OR probiotic OR bifidobacteria OR *Lactobacillus* OR *Propionibacterium* OR *Lactococcus* OR *Oenococcus* OR *Bacterioides* OR *Firmicutes*) AND (disease OR health OR growth OR dysbiosis OR infection OR CID OR "common infectious disease" OR disorder OR "host behavior" OR development OR "common cold" OR flu OR "Rota virus" OR rotavirus OR immune OR inflammatory OR inflammation OR metabolism OR metabolic OR colonization OR establish OR "gut microbiota composition" OR pediatric) AND (milk OR "infant diet" OR weaning OR feed OR nutrient OR supplement OR Supplemental OR "milk-based formula" OR nutrition OR nourish OR nutriment OR regime OR "environmental factor" OR prebiotic OR probiotic OR "probiotic consumption" OR symbiotic) AND (infancy OR infant OR baby OR babies OR neonate OR newborn OR offspring OR "early life" OR "early-life" OR "1000 day" OR "1000-day" OR "One thousand day" OR "post-natal" OR postnatal OR "post natal" OR weaning OR "complementary feeding" OR "complementary food" OR maternal OR "pre-schooler" OR "pre schooler" OR preschooler OR childhood OR child OR children OR "after birth" OR pediatric)) AND (infant OR neonate OR baby OR babies OR newborn OR offspring OR "pre-schooler" OR "pre schooler" OR preschooler OR infancy OR child OR "early childhood" OR "early life" OR weaning OR "after birth") AND ("infant microbiome" OR "fecal microbiota" OR microbiome OR microbiota OR microflora OR "gut-microflora" OR "gut-microbiome" OR "gut-microbiota" OR "Gastrointestinal Microbiome" OR enterotype OR "gut microbiome" OR probiotic OR symbiotic OR "beneficial bacteria" OR "promoting bacteria" OR "beneficial microbe" OR "promoting microbe" OR "beneficial microorganism" OR "promoting microorganism" OR "protective microorganism" OR "protective microorganism")) NOT (mice OR mouse OR "*Mus musculus*" OR rodent OR rat OR rats OR *Rattus* OR monkey OR "non-human primate" OR *Gorilla* OR pig OR pigs OR *Sus* OR sow OR sows OR calves OR calf OR *Canis* OR dog OR dogs OR insect OR insects OR "honey bees" OR A*p*is OR *Drosophila* OR fishes OR cat OR cats OR *Feline* OR lamb OR lambs OR *Ovis* OR "plant microbio" OR broiler OR *Caenorhabditis* OR rabbit OR bovine OR fungi OR shrimp OR "rainbow trout" OR *Oncorhynchus* OR chicken OR soil OR bear OR ruminal OR rumen OR pigeons OR zebrafish OR hens OR puppies OR piglet OR piglets OR pandas OR *Ailuropoda* OR steer OR steers OR mites OR "sea cucumber" OR Apostichopus OR goat OR goats OR "*in-vitro*" OR "*in vitro*" OR "*in vivo*"

OR "*in-vivo*" OR horses OR equine OR colt OR colts OR foal OR nag OR nags OR "pre-term" OR preterm OR "pre term"))) NOT (title:(mice OR mouse OR "*Mus musculus*" OR rodent OR rat OR rats OR *Rattus* OR monkey OR "non-human primate" OR *Gorilla* OR pig OR pigs OR *Sus* OR sow OR sows OR calves OR calf OR *Canis* OR dog OR dogs OR insect OR insects OR "honey bees" OR *Apis* OR *Drosophila* OR fishes OR cat OR cats OR *Feline* OR lamb OR lambs OR Ovis OR "plant microbio" OR broiler OR *Caenorhabditis* OR rabbit OR bovine OR fungi OR shrimp OR "rainbow trout" OR *Oncorhynchus* OR chicken OR soil OR bear OR ruminal OR rumen OR pigeons OR zebrafish OR hens OR puppies OR piglet OR piglets OR pandas OR *Ailuropoda* OR steer OR steers OR mites OR "sea cucumber" OR *Apostichopus* OR goat OR goats OR "*in-vitro*" OR "*in vitro*" OR "*in vivo*" OR "*in-vivo*" OR horses OR equine OR colt OR colts OR foal OR nag OR nags OR "pre-term" OR preterm OR "pre term"))

(b) infant nutrition and microbiome metabolism: groups of keywords were collected and intersected to select abstracts about the effects of infant nutrition on its gut microbiota, occurring during the weaning period.

(title,abstract:((((microbe OR microbial OR bacterial OR gut OR microbiome OR "microbial genome" OR symbiotic OR microbiota OR microflora OR "Gastrointestinal microbiome" OR metanalysis OR "meta-analysis" OR metagenome OR "meta-genome" OR archea OR probiotic OR bifidobacteria OR *Lactobacillus* OR *Propionibacterium* OR *Lactococcus* OR *Oenococcus* OR *Bacterioides* OR *Firmicutes*) AND (disease OR health OR growth OR dysbiosis OR infection OR CID OR "common infectious disease" OR disorder OR "host behavior" OR development OR "common cold" OR flu OR "Rota virus" OR rotavirus OR immune OR inflammatory OR inflammation OR metabolism OR metabolic OR colonization OR establish OR "gut microbiota composition" OR pediatric) AND (milk OR "infant diet" OR weaning OR feed OR nutrient OR supplement OR Supplemental OR "milk-based formula" OR nutrition OR nourish OR nutriment OR regime OR "environmental factor" OR prebiotic OR probiotic OR "probiotic consumption" OR symbiotic) AND (infancy OR infant OR baby OR babies OR neonate OR newborn OR offspring OR "early life" OR "early-life" OR "1000 day" OR "1000-day" OR "One thousand day" OR "post-natal" OR postnatal OR "post natal" OR weaning OR "complementary feeding" OR "complementary food" OR maternal OR "pre-schooler" OR "pre schooler" OR preschooler OR childhood OR child OR children OR "after birth" OR pediatric)) AND ("bifidus factor" OR prebiotic OR FOS OR fructooligosaccharide OR oligofructose OR GOS OR galactooligosaccharide OR glycans OR "4-galactooligosaccharide" OR "diet therap" OR "human milk oligosaccharide" OR HMO OR butyrate OR acetate OR propionate OR lactate OR succinate OR FOS OR fructooligosaccharide OR oligofructose OR GOS OR galactooligosaccharide OR "human milk") AND ((infant OR baby OR neonate OR newborn OR offspring OR "early-lif"e OR "early life" OR 1000 day OR postnatal OR weaning OR "post-natal" OR "pre-schooler" OR "pre schooler" OR preschooler OR childhood OR child OR children OR pediatric) OR ("postnatal factor" OR "infant nutrition" OR "infant food" OR "solid food" OR "infant diet" OR feeding OR weaning OR "Complementary feeding" OR "complementary food" OR "milk human" OR "human milk" OR "infant formula" OR "infant microbiome" OR "breast feed" OR "breast fed" OR "breast-feed" OR "breast-fed" OR "formula feed" OR "formula-feed" OR "formula-fed" OR "formula fed" OR "Supplemental feeding" OR "breast feeding" OR "breast-feeding") OR (diet OR dietary OR "fecal microbiota" OR "developing infant gut" OR "microbial change" OR "microbial shift")) AND (("infant microbiome" OR "healthy interaction" OR "host interaction" OR "infant development" OR "early life colonization" OR "early colonization" OR "Microbiological Phenomena" OR "infant-stool" OR "infant stool" OR probiotic OR host OR symbiotic) OR ("gastrointestinal microbiome" OR host OR microbiome OR microbiota OR microflora OR "gut-microflora" OR "gut-microbiome" OR "Gastrointestinal Microbiome" OR

enterotype OR "gut microbiome" OR "microbial composition" OR "microbiota composition" OR "infant microbiome" OR "intestinal microorganism" OR "neonatal microbiome" OR "neonatal gut" OR "neonatal microbiota" OR "children gut" OR "children microbiota" OR "children microbiome" OR symbiotic probiotic) OR ("beneficial bacteria" OR "beneficial microbe" OR "healthy bacteria" OR "healthy microbe" OR "healthy microorganism" OR "protective bacteria" OR "protective microorganism" OR "promoting bacteria" OR "promoting microorganism" OR "promoting microbe" OR probiotic OR symbiotic))) NOT (mice OR mouse OR "*Mus musculus*" OR rodent OR rat OR rats OR *Rattus* OR monkey OR "non-human primate" OR *Gorilla* OR pig OR pigs OR *Sus* OR sow OR sows OR calves OR calf OR *Canis* OR dog OR dogs OR insect OR insects OR "honey bees" OR *Apis* OR *Drosophila* OR fishes OR cat OR cats OR *Feline* OR lamb OR lambs OR *Ovis* OR "plant microbio" OR broiler OR *Caenorhabditis* OR rabbit OR bovine OR fungi OR shrimp OR "rainbow trout" OR *Oncorhynchus* OR chicken OR soil OR bear OR ruminal OR rumen OR pigeons OR zebrafish OR hens OR puppies OR piglet OR piglets OR pandas OR *Ailuropoda* OR steer OR steers OR mites OR "sea cucumber" OR *Apostichopus* OR goat OR goats OR "*in-vitro*" OR "*in vitro*" OR "*in vivo*" OR "*in-vivo*" OR horses OR equine OR colt OR colts OR foal OR nag OR nags OR "pre-term" OR preterm OR "pre term"))) NOT (title:(mice OR mouse OR "*Mus musculus*" OR rodent OR rat OR rats OR *Rattus* OR monkey OR "non-human primate" OR *Gorilla* OR pig OR pigs OR *Sus* OR sow OR sows OR calves OR calf OR *Canis* OR dog OR dogs OR insect OR insects OR "honey bees" OR *Apis* OR *Drosophila* OR fishes OR cat OR cats OR *Feline* OR lamb OR lambs OR *Ovis* OR "plant microbio" OR broiler OR *Caenorhabditis* OR rabbit OR bovine OR fungi OR shrimp OR "rainbow trout" OR *Oncorhynchus* OR chicken OR soil OR bear OR ruminal OR rumen OR pigeons OR zebrafish OR hens OR puppies OR piglet OR piglets OR pandas OR *Ailuropoda* OR steer OR steers OR mites OR "sea cucumber" OR *Apostichopus* OR goat OR goats OR "*in-vitro*" OR "*in vitro*" OR "*in vivo*" OR "*in-vivo*" OR horses OR equine OR colt OR colts OR foal OR nag OR nags OR "pre-term" OR preterm OR "pre term"))

(c) beneficial bacteria supporting the development of the infants immune system: groups of keywords were collected and intersected to select abstracts of studies that investigated the influence of the gut microbiota on the development of the immune system in infants.

(title,abstract:((((microbe OR microbial OR bacterial OR gut OR microbiome OR "microbial genome" OR symbiotic OR microbiota OR microflora OR "Gastrointestinal microbiome" OR metanalysis OR "meta-analysis" OR metagenome OR "meta-genome" OR archea OR probiotic OR bifidobacteria OR *Lactobacillus* OR *Propionibacterium* OR *Lactococcus* OR *Oenococcus* OR *Bacterioides* OR *Firmicutes*) AND (disease OR health OR growth OR dysbiosis OR infection OR CID OR "common infectious disease" OR disorder OR "host behavior" OR development OR "common cold" OR flu OR "Rota virus" OR rotavirus OR immune OR inflammatory OR inflammation OR metabolism OR metabolic OR colonization OR establish OR "gut microbiota composition" OR pediatric) AND (milk OR "infant diet" OR weaning OR feed OR nutrient OR supplement OR Supplemental OR "milk-based formula" OR nutrition OR nourish OR nutriment OR regime OR "environmental factor" OR prebiotic OR probiotic OR "probiotic consumption" OR symbiotic) AND (infancy OR infant OR baby OR babies OR neonate OR newborn OR offspring OR "early life" OR "early-life" OR "1000 day" OR "1000-day" OR "One thousand day" OR "post-natal" OR postnatal OR "post natal" OR weaning OR "complementary feeding" OR "complementary food" OR maternal OR "pre-schooler" OR "pre schooler" OR preschooler OR childhood OR child OR children OR "after birth" OR pediatric)) AND (("infant microbiome" OR "healthy interaction" OR "host interaction" OR "infant development" OR "early life colonization" OR "early colonization" OR "Microbiological Phenomena" OR "infant-stool" OR "infant stool" OR probiotic OR host OR symbiotic) OR ("beneficial bacteria" OR "beneficial microbe" OR

"healthy bacteria" OR "healthy microbe" OR "healthy microorganism" OR "protective bacteria" OR "protective microorganism" OR "promoting bacteria" OR "promoting microorganism" OR "promoting microbe" OR probiotic OR symbiotic)) AND ((diet OR dietary OR "fecal microbiota" OR "developing infant gut" OR "microbial change" OR "microbial shift") OR ("beneficial bacteria" OR "beneficial microbe" OR "healthy bacteria" OR "healthy microbe" OR "healthy microorganism" OR "protective bacteria" OR "protective microorganism" OR "promoting bacteria" OR "promoting microorganism" OR "promoting microbe" OR probiotic OR symbiotic)) AND ("Immune gene" OR cytokine OR antibod OR immunoglobulin OR "Immune Evasion" OR Immunomodulation OR "Maternally-Acquired Immunity" OR "maternal immunity" OR "innate immunity" OR Immun OR "immune-mediated disease" OR "immune mediated disease" OR monocyte OR immunonutrition OR "immuno nutrition" OR "Growth and Development" OR "paediatric dysbiosis" OR "Infant Nutritional Physiological Phenomena" OR immuno OR "antimicrobial protein")) NOT (mice OR mouse OR "*Mus musculus*" OR rodent OR rat OR rats OR *Rattus* OR monkey OR "non-human primate" OR *Gorilla* OR pig OR pigs OR *Sus* OR sow OR sows OR calves OR calf OR *Canis* OR dog OR dogs OR insect OR insects OR "honey bees" OR *Apis* OR *Drosophila* OR fishes OR cat OR cats OR *Feline* OR lamb OR lambs OR *Ovis* OR "plant microbio" OR broiler OR *Caenorhabditis* OR rabbit OR bovine OR fungi OR shrimp OR "rainbow trout" OR *Oncorhynchus* OR chicken OR soil OR bear OR ruminal OR rumen OR pigeons OR zebrafish OR hens OR puppies OR piglet OR piglets OR pandas OR *Ailuropoda* OR steer OR steers OR mites OR "sea cucumber" OR *Apostichopus* OR goat OR goats OR "*in-vitro*" OR "*in vitro*" OR "*in vivo*" OR "*in-vivo*" OR horses OR equine OR colt OR colts OR foal OR nag OR nags OR "pre-term" OR preterm OR "pre term"))) NOT (title:(mice OR mouse OR "*Mus musculus*" OR rodent OR rat OR rats OR *Rattus* OR monkey OR "non-human primate" OR *Gorilla* OR pig OR pigs OR *Sus* OR sow OR sows OR calves OR calf OR Canis OR dog OR dogs OR insect OR insects OR "honey bees" OR *Apis* OR *Drosophila* OR fishes OR cat OR cats OR *Feline* OR lamb OR lambs OR *Ovis* OR "plant microbio" OR broiler OR *Caenorhabditis* OR rabbit OR bovine OR fungi OR shrimp OR "rainbow trout" OR *Oncorhynchus* OR chicken OR soil OR bear OR ruminal OR rumen OR pigeons OR zebrafish OR hens OR puppies OR piglet OR piglets OR pandas OR *Ailuropoda* OR steer OR steers OR mites OR "sea cucumber" OR *Apostichopus* OR goat OR goats OR "*in-vitro*" OR "*in vitro*" OR "*in vivo*" OR "*in-vivo*" OR horses OR equine OR colt OR colts OR foal OR nag OR nags OR "pre-term" OR preterm OR "pre term"))

(d) beneficial bacteria preventing infections in infants: groups of keywords were collected and intersected to select abstracts of studies that investigated the role of beneficial gut bacteria in preventing infections in infants.

(title,abstract:((((microbe OR microbial OR bacterial OR gut OR microbiome OR "microbial genome" OR symbiotic OR microbiota OR microflora OR "Gastrointestinal microbiome" OR metanalysis OR "meta-analysis" OR metagenome OR "meta-genome" OR archea OR probiotic OR bifidobacteria OR *Lactobacillus* OR *Propionibacterium* OR *Lactococcus* OR *Oenococcus* OR *Bacterioides* OR *Firmicutes*) AND (disease OR health OR growth OR dysbiosis OR infection OR CID OR "common infectious disease" OR disorder OR "host behavior" OR development OR "common cold" OR flu OR "Rota virus" OR rotavirus OR immune OR inflammatory OR inflammation OR metabolism OR metabolic OR colonization OR establish OR "gut microbiota composition" OR pediatric) AND (milk OR "infant diet" OR weaning OR feed OR nutrient OR supplement OR Supplemental OR "milk-based formula" OR nutrition OR nourish OR nutriment OR regime OR "environmental factor" OR prebiotic OR probiotic OR "probiotic consumption" OR symbiotic) AND (infancy OR infant OR baby OR babies OR neonate OR newborn OR offspring OR "early life" OR "early-life" OR "1000 day" OR "1000-day" OR "One thousand day"

OR "post-natal" OR postnatal OR "post natal" OR weaning OR "complementary feeding" OR "complementary food" OR maternal OR "pre-schooler" OR "pre schooler" OR preschooler OR childhood OR child OR children OR "after birth" OR pediatric)) AND (("infant microbiome" OR "healthy interaction" OR "host interaction" OR "infant development" OR "early life colonization" OR "early colonization" OR "Microbiological Phenomena" OR "infant-stool" OR "infant stool" OR probiotic OR host OR symbiotic) OR ("beneficial bacteria" OR "beneficial microbe" OR "healthy bacteria" OR "healthy microbe" OR "healthy microorganism" OR "protective bacteria" OR "protective microorganism" OR "promoting bacteria" OR "promoting microorganism" OR "promoting microbe" OR probiotic OR symbiotic)) AND ((diet OR dietary OR "fecal microbiota" OR "developing infant gut" OR "microbial change" OR "microbial shift") OR ("beneficial bacteria" OR "beneficial microbe" OR "healthy bacteria" OR "healthy microbe" OR "healthy microorganism" OR "protective bacteria" OR "protective microorganism" OR "promoting bacteria" OR "promoting microorganism" OR "promoting microbe" OR probiotic OR symbiotic)) AND ("beta-defensin" OR defensin OR inflammation OR inflammatory OR vaccin OR "secretory IgA and TNF-a" OR infection OR "Bacterial Infection" OR "Host-Pathogen Interaction" OR "common infectious disease" OR colitis OR enterocolitis OR Colitis OR enterocolitis OR influenza OR flu OR CID OR "immunity infection")) NOT (mice OR mouse OR "*Mus musculus*" OR rodent OR rat OR rats OR *Rattus* OR monkey OR "non-human primate" OR *Gorilla* OR pig OR pigs OR Sus OR sow OR sows OR calves OR calf OR *Canis* OR dog OR dogs OR insect OR insects OR "honey bees" OR *Apis* OR Drosophila OR fishes OR cat OR cats OR *Feline* OR lamb OR lambs OR *Ovis* OR "plant microbio" OR broiler OR Caenorhabditis OR rabbit OR bovine OR fungi OR shrimp OR "rainbow trout" OR Oncorhynchus OR chicken OR soil OR bear OR ruminal OR rumen OR pigeons OR zebrafish OR hens OR puppies OR piglet OR piglets OR pandas OR *Ailuropoda* OR steer OR steers OR mites OR "sea cucumber" OR *Apostichopus* OR goat OR goats OR "*in-vitro*" OR "*in vitro*" OR "*in vivo*" OR "*in-vivo*" OR horses OR equine OR colt OR colts OR foal OR nag OR nags OR "pre-term" OR preterm OR "pre term"))) NOT (title:(mice OR mouse OR "*Mus musculus*" OR rodent OR rat OR rats OR *Rattus* OR monkey OR "non-human primate" OR *Gorilla* OR pig OR pigs OR *Sus* OR sow OR sows OR calves OR calf OR *Canis* OR dog OR dogs OR insect OR insects OR "honey bees" OR *Apis* OR *Drosophila* OR fishes OR cat OR cats OR *Feline* OR lamb OR lambs OR *Ovis* OR "plant microbio" OR broiler OR *Caenorhabditis* OR rabbit OR bovine OR fungi OR shrimp OR "rainbow trout" OR *Oncorhynchus* OR chicken OR soil OR bear OR ruminal OR rumen OR pigeons OR zebrafish OR hens OR puppies OR piglet OR piglets OR pandas OR *Ailuropoda* OR steer OR steers OR mites OR "sea cucumber" OR *Apostichopus* OR goat OR goats OR "*in-vitro*" OR "*in vitro*" OR "*in vivo*" OR "*in-vivo*" OR horses OR equine OR colt OR colts OR foal OR nag OR nags OR "pre-term" OR preterm OR "pre term"))

Supplemental Information 4

Dictionaries used for relationship and co-mention identification in abstracts and full-texts from the PubMed IDs collection.

For each term in dictionaries, we considered both synonyms and most common separators between specific words. The operator = was used for exact matching, otherwise a more flexible orthographic variant search of the words into texts was applied.

(i) immunity-related terms

α-1 antitrypsin, α 1 antitrypsin, α1 antitrypsin, α1-antitrypsin, α 1-antitrypsin, α1antitrypsin, C Reactive Protein, C-Reactive Protein, =CRP, immunoglobulin, Immunoglobulin A, =IgA, Secretory Immunoglobulin A, =SIgA, Tumor Necrosis Factor-alpha, Tumor Necrosis Factor alpha, =TNFα, =TNF α, =TNF-α, Immunoglobulin G, =IgG, cytokines, immunity biomarker, Immune marker, Immunological marker, immune, immunity, =IFN-γ, =IFN γ, =IFNγ, Interferon gamma, Interferon-gamma, =IL-4, =IL 4, =IL4, interleukin 4, interleukin-4, =IL-6, =IL 6, =IL6, interleukin 6, interleukin-6, =IL-1β, =IL 1β, =IL1β, =IL1 β, =IL-1 β, =IL 1 β, interleukin 1β, interleukin-1β, interleukin-1 β, interleukin 1 β, chemokine, immunostimulant, immuno stimulant, immuno-stimulant, Immunotherapy, Immuno-therapy, Immuno therapy, Immunogenetic, Immuno-genetic, Immuno genetic, immunonutrition, immuno nutrition, immuno-nutrition, immunosuppression, immuno-suppression, immuno suppression, autoimmune disorder, auto-immune disorder, auto immune disorders, immunologic diseases, immunologic abnormalities, immunoregulatory abnormalities, immuno regulatory abnormalities, immuno-regulatory abnormalities, immune dysregulation, immune deficient, immunedeficient, immune-deficient, immunological tolerance, immunologic diseases, immunological diseases, defensin, α-defensin, α defensin, β-defensin, β defensin, =HBD-2, =HBD 2, =HBD2, =HNP 1, =HNP-1, Cathelicidin, =LL-37, =hCAP-18, =hCAP 18, cationic antimicrobial protein, hCAP, neutrophil peptide, neutrophil defensin, Toll Like Receptor, Toll Like Receptor-4, Toll Like Receptor 4, =TLR, =TLR-4, =TLR4, =TLR 4, macrophages, natural killer cells, NK Cells, =NK, antigen-specific cytotoxic, antigen specific, antigen-specific, =T-lymphocytes, =T lymphocytes, Tlymphocytes, cytotoxic T-lymphocytes, lymphocytes, cytotoxic Tlymphocytes, cytotoxic T lymphocytes, Immunologic Cytotoxicity, Cytotoxicity, cytotoxic.

(ii) infant infection and disease related terms

abdominal distension, abdominal distension and bilious vomiting, abdominal pain, acute asthma, acute asthma exacerbations, acute colitis, Acute diarrhea, acute diarrhea and acute respiratory tract infections, acute diarrheal diseases, acute febrile infections, acute gastro-enteritis, acute gastroenteritis diarrhea, acute gastrointestinal inflammation, acute GI-GVHD, acute infantile diarrhea, acute infection, acute infectious diarrhea, acute lower respiratory tract infection, acute otitis, acute otitis media, acute pharyngotonsillitis, acute respiratory infection, acute rotavirus diarrhea, acute tonsillitis, acute ulcerative colitis, Acute Upper Respiratory Infection, adenoiditis, airway infections, airways disease, allergic airways disease, allergic and inflammatory disorders, allergic asthma, allergic colitis, allergic condition, allergic conjunctivitis, allergic dermatitis, Allergic gastroenteritis and colitis, allergic inflammation, allergic proctocolitis, allergic rhinitis, allergic rhinoconjunctivitis, aseptic meningitis, asthma, bacillary dysentery, Bacteremic infection, bacterial diarrhea, bacterial dysbiosis, bacterial gastroenteritis, Bacterial infection, bacterial intestinal disease, Bacterial skin diseases, bacterial UTI, =UTI, bacterial, viral and respiratory infections, Bacterimia, bacterimica, bilious vomiting, C. difficile associated , C. difficile

associated disease, C. difficile infection, C. difficile-associated diseases, Campylobacter enteritis, Campylobacter infections, Candida infection, candidiasis, caries, chlamydial infections, Cholangitis, Chronic infection, clostridial infections, clostridial intestinal infections, Clostridium difficile associated , Clostridium difficile associated disease, Clostridium difficile infection, Clostridium difficile-associated disease, Clostridium infections, coeliac disease, cold, cold infections, coli infections, colitis, common cold, common infection disease, common infectious disease, conjunctivitis, dental caries, dermatitis, diarrhea, diarrhea and respiratory tract infections, diarrheal, diarrheal diseases, diarrhoeal illness, Diphtheria, disbiosis, Dysbiosis, dysentery, E. coli infections, E.coli infections, ear infections, endocarditis, enteric infection, enteritis, Enterocolitis, enteropathy, febrile infections, Flu, food allergy, food hypersensitivity, fungal infection, gastro enteritis, gastro enteritis diarrhea, gastroenteritis, gastro-enteritis, gastroenteritis diarrhea, gastro-enteritis diarrhea, Gastrointestinal Diseases, gastrointestinal inflammation, GI infection, GI inflammation, giardiasis, gut inflammation, Haemophilus, Helicobacter infection, inflammatory disorders, influenza, Irritable Bowel Syndrome, laryngitis, lower respiratory tract infection, meningitis, mumps, Mycoplasmosis, Nosocomial, nosocomial diarrhea, Nosocomial infections, Osteomyelitis, otitis, paediatric disorders, paediatric dysbiosis, Pertussis, pharyngitis, pharyngotonsillitis, pneumonia, proctocolitis, respiratory infection, respiratory syncytial virus, respiratory tract infections, rhinitis, rhinoconjunctivitis, rhino-conjunctivitis, rotavirus diarrhea, Rotavirus infections, rubella, Sepsis, Staphylococcal infections, Tetanus, thrush, tonsillitis, tracheitis, ulcerative colitis, Upper Respiratory Infection, urinary infections, urinary tract infections, viral infection, Whooping Cough, =CID, =GI-GVHD

(iii) chemical terms

lacto- N -fucopentaose, =2FL, =2'-FL, =2'-O-fucosyllactose, =n 3-Fatty Acid, =n 3-Oil, =n 3-Polyunsaturated Fatty Acid, =n 3-PUFA, =n-3 Fatty Acid, =n-3 Oil, =n-3 Polyunsaturated Fatty Acid, =n-3 PUFA, 1,2-benzenedicarboxylic, 2,3 dihydroxy benzoic acid, 25-hydroxy-vitamin D, 2fucosyllactose, 2'-fucosyllactose, 2-fucosyllactose, 2'Fucosyllactose , 2-hydroxyisovalerate, 2'-O-fucosyllactose, 3-aminoisobutyrate, aminoisobutyrate, 3-difucosyllactose, difucosyllactose, 3-fucosyllactose, fucosyllactose, 3-indoleacetate, indoleacetate, acetylgalactosaminyl, 3'-N-acetylgalactosaminyl-galactose, 3-N-acetylgalactosaminyl-galactose, 3'-N-acetylglucosaminyl-mannose, 3-O-methylglucose, 3-phenyllactic acids, 3'-sialyllactose, sialyllactose, 3'-sialyllactose, 4' -galactobiose, galactobiose, 4,5-dihydroxy-2,3-pentanedione, 4-galactobiose, 4-galactooligosaccharide, galactooligosaccharide, 4-galactosyllactose, galactosyllactose, 4-galactotriose, galactotriose, 4-hydroxythreonine-4-phosphate, hydroxythreonine, 6'-galactobiose, 6-phosphogluconate, phosphogluconate, 6'-sialyllactose, Acetate, acetic, butyric, lactic, acetic and butyric acids, acetic and lactic acids, acetogens, acetoin, acetyl CoA, Acetyl glucosamine, Acetyl Neuraminic, Acetylglucosamine, Acetyl-glucosamine, acetylneuraminic, acetyl-neuraminic, alpha lipoic acid, alpha1,2-fucosyl, alpha1,3-fucosyl, alpha-lactalbumin, arabinogalactan, arabinose, arabinoxylan, arginine, Arginine Hydrochloride, b1-3- and b1-6-galactosides, b-d-galactosides, beta-carotene, betaD glucosamine, beta-D glucosamine, betaD-glucosamine, beta-D-glucosamine, beta-galactoside, BetaGlcNAc, beta-palmitic acid, b-galactoside, bile acid, bile salt, bilirubin, b-N-acetylhexosaminidase, butyrate, Butyrate , Butyric Acids, butyrophilin, calprotectin, carbohydrate, carnitine, cholic acid, coenzyme B12, coenzyme Q10, cysteamine, cysteamine bitartrate, D- and L- lactate, d-glucose 6-phosphate, d -glucose 6 -phosphate, glucose 6-phosphate, glucose 6 phosphate, decosahexenoic acid, D-galactose, galactose, D-Glutamine, dihomo-gamma-linolenic acids, linolenic acids, dihomolinolenoyl-CoA, dihydroxyvalerolactone sulphate, disaccharides, disialyl-lacto-N-tetraose, D-lactate, DL-Arginine Acetate, Arginine Acetate, DocosaHexaenoic Acid, dTDP-4-dehydrorhamnose, dehydrorhamnose, D-xylose, xylose, ellagic acid, epicatechin, epigallocatechin, epigallocatechin gallate, fatty acid, fructo

oligosaccharide, fructooligosaccharide, fructooligosaccharide, fructo-oligosaccharide, fructose, fructose-6-phosphate, fucose, fucosyl, fucosyl disialyl, fucosyl lactose, fucosyldisialyl, fucosyl-disialyl, fucosyl-disialyl-lacto-N-hexaose, fucosyllactose, galacto oligosaccharide, galacto-N-biose, galactooligosaccharide, galactooligosaccharide, galacto-oligosaccharide, galactose-1-phosphate, galactoside, galactotriose, Galbeta1 3GlcNAc, Galbeta1,3GlcNAc, Galbeta1-3GlcNAc, ganglioside, =GlcNAc, =3GlcNAc, GlcNAc beta, GlcNAc-beta, gluco mannan, glucomannan, gluco-mannan, glucopyranose, glucosamine, Glucose-6-phosphate, glutamine, =HMO, human milk, human milk oligosaccharide, inulin type fructans, inulin-type fructans, Lactate, lactic acid, Lacto N difucohexaose, lacto- N -difucohexaose, lacto N fucopentaose 2, lacto N fucopentaose II, lacto N hexaose, Lacto N neotetraose, lacto- N -neotetraose, lacto- N -neotetraose, Lacto N tetraose, lacto- N -tetraose, lacto- N -tetraose, Lacto N-biose, lacto N-fucopentaose 2, lacto N-fucopentaose II, lacto N-hexaose, Lacto N-neotetraose, Lacto N-tetraose, Lacto-N biose, Lacto-N difucohexaose, lacto-N -difucohexaose, lacto-N fucopentaose 2, lacto-N fucopentaose II, lacto-N hexaose, Lacto-N neotetraose, Lacto-N tetraose, Lacto-N-biose, Lacto-N-biose, lacto-N-difucohexaose, lacto-N-fucopentaose, lacto-N-fucopentaose 2, lacto-N-fucopentaose II, lacto-N-hexaose, Lacto-N-neotetraose, Lacto-N-neotetraose, lacto-N-tetraose, Lacto-N-tetraose, lacto-N-triose, Lactose, lactose factor, Lactotetraose, Lactotetraose-HSA, lactulose, L-Arginine, L-fucose, L-Glutamine, =LND, =LNDFH, Lnfp 2, Lnfp II, Lnfp2, Lnfp-2, LnfpII, Lnfp-II, maltodextrin, mannan, mannose, mannuronic acid, mannuronic, N Acetyl, N -acetyl glucosamine, N -acetylgalactosamine, N -acetylglucosamine, N -acetyllactosamine, N -acetylneuraminic acid, N -glycoylneuraminic acid, n-3 polyunsaturated fatty acids, N-Acetyl Neuraminic Acid, N-Acetyl-beta-D-glucosamine, N-acetyl-cysteine, N-acetylgalactosamine, N-acetylglucosamine, N-acetyl-glucosamine, N-acetylneuraminic acid, N-Acetyl-Neuraminic Acid, N-ethylmaleimide, =Neu5Ac, =NeuAc, Neurofurans (NeuFs), Neurofurans, =NeuFs, O-desmethylangolensin (O-DMA), O-desmethylangolensin, O desmethylangolensin, desmethylangolensin, =O-DMA, oligofructose, oligosaccharides, Omega 3 Fatty Acid, Omega3 Fatty Acid, omega-3 fatty acid, Omega-3 Fatty Acid, Polydextrose, polyethylene glycol, polyphenol, Polysaccharides, propionate, propionic acid, pyruvate, Resveratrol, Retinol, retinyl palmitate, palmitate, r-Lactotetraose-HSA, Lactotetraose, S-adenosylhomocysteine, adenosylhomocysteine, =SCFA, short chain fatty acid, short-chain fatty acid, sialic acid, Sialic-acid, sialylated lacto- N -tetraose, sialylatedN, sialylatedN-glycans, sialyllactose, sialyloligosaccharide, sodium taurocholate, sorbitol, sphingolipids, sphingomyelin, S-ribosyl-homocysteine, succinate, succinic acid, taurodeoxycholic acid, trans-beta-galacto-oligosaccharides, tri-saccharides, trisaccharides, saccharides, turanose, tyramine, ursodeoxycholic acid, ursodeoxycholic, vitamin, xylobiose, xylooligosaccharide

Supplemental Information 5

List of foods (209) that were excluded from the whole list.

Adobo, Akutaq, Arabica coffee, Arepa, Ascidians, Bagel, Baked potato, Beer, Biscuit, Breakfast cereal, Breakfast sandwich, Burrito, Butterfat, Butter, Butter substitute, Cake, Candy bar, Chewing gum, Chanterelle, Chili, Chimichanga, Chocolate, Chocolate mousse, Chocolate spread, Cloud ear fungus, Cocoa butter, Cocktail, Coffee, Coffee mocha, Coffee substitute, Cold cut, Corn grits, Cornbread, Common mushroom, Conch, Cooking oil, Corn chip, Corn salad, Cracker, Cream, Cream substitute, Crisp bread, Curry powder, Dripping, Dulce de leche, Dumpling, Egg roll, Egg substitute, Empanada, Enchilada, Energy drink, Enokitake, Falafel, Fish burger, Focaccia, French toast, Frozen yogurt, Fruit gum, Fruit juice, Fruit preserve, Fruit salad, Fruit-flavor drink, Frybread, Fudge, Gelatin, Gelatin dessert, Gin, Grape wine, Hamburger, Horchata, Hot chocolate, Hot dog, Hummus, Hushpuppy, Ice cream, Ice cream cone, Icing, Junket, Ketchup, Lasagna, Lard, Leather chiton, Leavening agent, Liquor, Macaroni and cheese, Madeira wine, Maitake, Margarine, Margarine-like spread, Marshmallow, Marzipan, Meat bouillon, Meatball, Meringue, Milkshake, Mixed nuts, Molasses, Morchella (Morel), Multigrain bread, Muskrat, Mustard, Nachos, Natto, Nougat, Nutritional drink, Oat bread, Oil palm, Opium poppy, Ostrich fern, Other alcoholic beverage, Other animal fat, Other beverage, Other bread product, Other candy, Other dish, Other fermented milk, Other fish product, Other frozen dessert, Other fruit product, Other meat product, Other pasta dish, Other sandwich, Other snack food, Other soy product, Other vegetable product, Oyster mushroom, Pan dulce, Pancake, Pastry, Pate, Pectin, Pepper (*C. annuum*), Pepper (*C. frutescens*), Pepper (Spice), Phyllo dough, Pie, Pie crust, Pie filling, Piki bread, Pizza, Popcorn, Port wine, Pot pie, Potato chip, Potato gratin, Potato puffs, Processed cheese, Pudding, Pupusa, Quesadilla, Raisin bread, Ravioli, Relish, Remoulade, Rice bread, Rice cake, Roe, Rum, Rye bread, Sake, Salad dressing, Salt, Sauce, Sausage, Scrapple, Sherry, Shiitake, Shortening, Snack bar, Snail, Soft drink, Soup, Sour cherry, Soy cream, Soy sauce, Spirit, Spread, Stew, Stuffing, Succotash, Sugar substitute, Sugar, White sucker, Syrup, Taco, Tallow, Tamale, Toffee, Topping, Tortilla, Tortilla chip, Tostada, Trail mix, Unclassified food or beverage, Vanilla, Vegetable juice, Vegetarian food, Vermouth, Vinegar, Vodka, Water, Waffle, Wasabi, Whey, Whelk, Whisky, Wonton wrapper, Ymer, Zwieback.

Supplemental Figure 1

A metabolic enrichment set analysis was carried out using the hypergeometric distribution implemented in bStyle [1]. The over-representation enrichment was performed for each member of the sub-community using organism-specific chemical compounds associated to their pathways automatically retrieved by the tool from the KEGG pathway database [2]. All strains were available into the database, except for *F. prausnitzii* M21 2 that was replaced by *F. prausnitzii SL3/*3 as it is belonging to the same phylogroup [3].

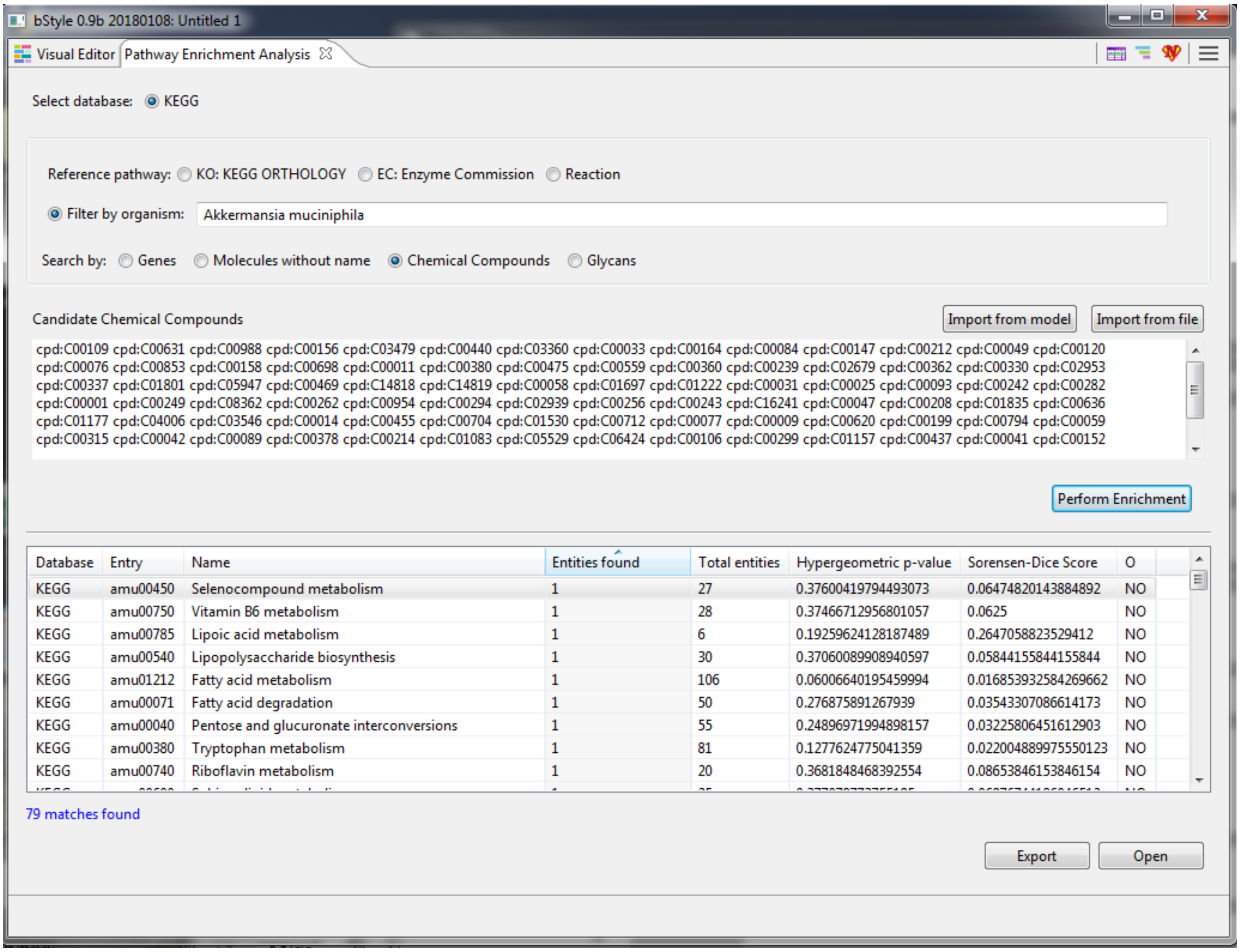

References

[1] Lombardo R, Priami C. Graphical Modeling Meets Systems Pharmacology. Gene Regul Syst Bio 2017;11:1177625017691937. doi:10.1177/1177625017691937.

[2] Ogata H, Goto S, Sato K, Fujibuchi W, Bono H, Kanehisa M. KEGG: Kyoto encyclopedia of genes and genomes. Nucleic Acids Res 1999;27:29–34. doi:10.1093/nar/27.1.29.

[3] Lopez-Siles M, Khan TM, Duncan SH, Harmsen HJM, Garcia-Gil LJ, Flint HJ. Cultured Representatives of Two Major Phylogroups of Human Colonic Faecalibacterium prausnitzii Can Utilize Pectin, Uronic Acids, and Host-Derived Substrates for Growth. Appl Environ Microbiol 2012;78:420–8. doi:10.1128/AEM.06858-11.
